# A multi objective optimization framework for smart parking using digital twin pareto front MDP and PSO for smart cities

**DOI:** 10.1038/s41598-025-91565-0

**Published:** 2025-03-05

**Authors:** Dinesh Sahu, Priyanshu Sinha, Shiv Prakash, Tiansheng Yang, Rajkumar Singh Rathore, Lu Wang

**Affiliations:** 1 SCSET, Bennett University, Plot Nos 8, 11, TechZone 2, Greater Noida, Uttar Pradesh 201310 India; 2 Department of Electronics and Communication, University of Allahabad, Prayag Raj, Uttar Pradesh India; 3 University of South Wales, Pontypridd, UK; 4 Cardiff School of Technologies, Cardiff Metropolitan University, Cardiff, UK; 5 Xi’an Jiaotong-Liverpool University, Suzhou, China

**Keywords:** Smart Parking Systems, Digital Twin Technology, Multi-Objective Optimization, Pareto Front Optimization, Markov Decision Process (MDP), Particle Swarm Optimization (PSO), Resource Allocation, Smart Cities, Traffic Congestion Management, Energy-Efficient Parking Solutions, Security, Computer science, Information technology

## Abstract

Smart cities are designed to improve the quality of life by efficiently using resources and smart parking is an important part of this puzzle to help alleviate traffic congestion and efficiently address energy consumption and search time for parking spaces. However, existing parking management systems have issues with resource management, system scalability, and real-time dynamic changes. In response to these challenges, this paper proposes a Multi-Objective Optimization Framework for Smart Parking incorporating Digital Twin Technology, Pareto Front Optimization, Markov Decision Process (MDP), and Particle Swarm Optimization (PSO). Hence, the proposed framework utilizes Digital Twin whereby there is a generation of a virtual model of the existing parking infrastructure that can give a real-time prospective estimation of the entire system. The Pareto Front is then used for multi-objective optimization of the search domain, where the goal is to minimize the search time, use of energy, and traffic disruption, and maximize the availability of parking spaces. The MDP splits the resource allocation problem into a value function which can then model the real-time parking requests. Further, PSO refines the solutions found from the Pareto front for a globally superior distribution. The framework is evaluated using extensive simulations across multiple metrics: search time, energy, congestion level, scalability, and utilization. Evaluation outcomes also show that the proposed algorithm is better than Round Robin, Random Allocation, and Threshold Based algorithms in terms of 25% improvement in the search time, 18% better energy usage, and 30% less traffic congestion. This work has shown the prospects of combining hybrid optimization and real-time decision-making in the enhancement of parking management in smart cities for better efficiency in urban mobility.

## Introduction

Thus, smart cities have become a prospective solution to address the issues of urbanization by means of using IoT, AI, and digital twinning of cities’ functionality^1^. Among the green energy technological enablers, a smart transportation system is undoubtedly one of the main strategic pillars of smart cities, as it recognizes efficient mobility as a basic prerequisite for sustainable urban development. In the context of transportation, the management of parking lots is one of the biggest issues because it concerns traffic, energy consumption, and environmental aspects^2^. The conventional parking systems have various problems in implementing and sustaining themselves in areas experiencing rapid urbanization. Time to Search and Time to find a Vacancy continue to be a problem since the increase in traffic flow due to advancement in urbanization puts pressure on parking facilities as vehicle numbers surpass the available capacities. Research studies show that every 30 to 40% of urban traffic in cities such as New York, London, and Tokyo is a result of drivers in the outlook for parking space^3^, which leads to pointless car circulation, time-wasting, and more congestion in already crowded areas. Undue time spent seeking for parking space leads to increased fuel consumption thereby increasing energy wastage as well as carbon emissions. Research shows that poor parking management is partly to blame for additional greenhouse gas emissions ranging between 15 and 20 percent in car-commuting cities thus exacerbating climate change^4,5^. Another major problem identified as Resource Under-utilization and Inefficiency is another major problem, as traditional parking systems do not have real-time data connection and analysis capabilities. This leads to idle parking spaces while other zones get congested due to parking slots statically being assigned and rarely dynamically shuffled^6^. Finally, the Lack of Scalability entails that existing systems are incapable of expanding when the size of the city, the population as well as the number of vehicles on the roads is on the increase. Without sophisticated mechanisms for allocating and optimizing resources based on actual usage, existing infrastructure can become rigid and unable to cope with real-time change requests resulting in wasted resources and lower user satisfaction levels^7^. Solving these issues is crucial for designing better, more efficient, and sustainable parking structures.

As a result, next-generation parking solutions need to meet a few important goals to overcome the limitations of the classical parking systems. Reducing the amount of time that people spend searching for parking spaces is important to free time for drivers, enhance user experience, and decrease holders’ search time that causes traffic jams. The most effective solutions for increasing energy efficiency that will lead to changes of the unused car movement and fuel consumption will positively influence parking systems effectiveness from the energy waste perspective. Effective and proactive allocation and re-allocation of the available parking spaces ensures optimization of the resources available hence meeting clients’ demand in different zones. Also, it is the real-time decision-making feature whereby resource utilization can be adjusted in a short time when operating in dynamic urban settings^8^. To such an end, the applied advanced parking systems must employ tools for multi-objective optimization of the conflictive objectives including the above-mentioned search time, energy usage, impacts on traffic jams, and resource exploitation. However, for more effective scalable, adaptive, and intelligent approaches for urban parking systems, predictive and real-time decision-making techniques, such as Digital Twins, Markov Decision Processes (MDP), and global optimization such as Particle Swarm Optimization (PSO), need to be developed^9,10^.

Advanced technologies affect a revolutionary change in the existing parking systems by facilitating monitoring, optimization, and control. DT creates replicas of physical systems with which to evaluate the parking status, vehicular arrival/departure rate, and traffic patterns in real-time and in virtual environments. This capability enables city administrators to be able to predict demand and allocate resources according to prevailing patterns on a real-time basis^11^. Multi-Objective Optimization with Pareto Front deals with mixed goals in car parking management in urban areas, including search time and energy, as well as congestion. Pareto front optimization defines solutions that are optimal between these goals Pareto front optimization defines solutions that are optimal between these goals Pareto front optimization defines how it is possible to find trade-off solutions more effectively^12^. MDP models real-time parking allocation as a sequential decision-making problem and hence allows dynamic adaptation to changes in demand, supply, and traffic patterns within the parking spaces. Since MDP decides based on optimal values and functions defined with respect to some reward, it guarantees optimal performance especially in policy decisions, delay, congestion, etc^13^. Last but not least, the nature-inspired meta-heuristic algorithm, Particle Swarm Optimization (PSO) solves the large-scale, non-linear, multi-objective problems. When applied to parking systems, PSO optimizes the resource allocator by further improving Pareto-optimal solutions so as to guarantee global optimization and system optimization^14–16^. All these advanced technologies together solve the scalability, adaptability, and smartness issues that are usually found in traditional parking systems. Smart parking framework enhances efficiency through real-time optimization through both edge and cloud but remains exposed to security risks such as data theft, unauthorized access, or edge-cloud infrastructure attacks. Proper protection of transmission and storage of information associated with vehicles and users is considered critical.

In contrast to the previous works, this study proposes a novel framework that includes: the Digital Twin Technology, Pareto Front Optimization, Markov Decision Process (MDP), and Particle Swarm Optimization (PSO) to acquire real-time, adaptive and multi-objective optimization of the smart parking. This novel integration allows dynamic decisions in the decision-making process and global optimality for parking space allocation hence making this technique a better heuristic-based method.

### Motivation

With the help of Digital Twin Technology, Pareto Front Optimization, Markov Decision Process (MDP), and Particle Swarm Optimization (PSO), a complete solution for smart parking systems can be designed and implemented. Therefore, this research postulates an adaptive hybrid framework that focuses on addressing the multi-objective trade-offs and providing real-time decisions for dynamic parking resources management, higher energy efficiency, and increased sustainability. Also, it has an influence on actual traffic by eliminating the heavy concentration of traffic flow and finding the best routes for the cars in the environment of the large cities. The prospects and efficiency of the proposed hybrid solution eliminate the shortcomings of existing parking systems and meet the objectives of environmentally friendly and rational smart cities.

### Objective of research

This research presented here shall develop a Multi-Objective Optimization Framework for Smart Parking which employs the application of sophisticated simulation models for real-time parking resource allocation. Specifically, the framework aims to:

1. Develop a series of scenarios for a Digital Twin used in simulating parking lots by developing a realistic simulation of parking lots to monitor, analyze, and perform subsequent predictive assessments on.
2. Use Pareto Front Optimization to balance multiple objectives related to the search time the energy that is used and congestion levels.
3. Proactively simulate the allocation of parking using the Markov Decision Process (MDP) in order to modify the strategy for parking based on current demand.
4. Use PSO to improve the favorable global optimization of Pareto optima which would improve the efficiency of the whole system.

The identified algorithms are designed to employ an integrated predictive analytical based, optimization, and decision-making solution to provide an effective smart city managing the parking lot with efficiency, reduced congestion level, and scalability.

### Novelty of proposed framework

The focus of this research innovation is an integrated hybrid composed of the Digital Twin Technology, Pareto optimization, MDP, and PSO approaches. Specifically:

1. *Digital Twin Technology* Digital Twin (DT) provides a replicant of the real-world parking environment and allows prediction simulation in real-time. DT is different from typical IoT-based parking systems where embedded sensors supply data to determine the current and the next state of parking^10,11^.
2. *Pareto Front Optimization* Pareto-based multi-objective optimization balances trade-offs between conflicting goals: reducing the time, energy, and traffic required for search while at the same time increasing resource-space utilization^12^. This way, the approach effectively avoids compromising one or several goals at the cost of one or several others.
3. *Markov Decision Process (MDP)* MDP can represent the dynamic resource allocation as a series of states or stages, actions, and rewards. It makes it possible for the system to respond to real-time parking needs as they are required. It is also noteworthy that the reward function is developed to promote short-term and long-term system performance^13,14^.
4. *Particle Swarm Optimization (PSO)* For optimization of resource allocation on the parking network, the PSO is used to fine-tune the Pareto front solutions^6^. PSO applies well in large-scale optimization problems hence making it applicable in large complex urban areas.

Unlike the prior work that cannot offer real-time, scalable, and energy-efficient parking management, the proposed framework incorporates these swift and efficient methods to handle the parking complexities that exist in large and dense environments such as cities.

### Contribution

The key contributions of this paper are summarized as follows:

1. *Digital Twin-Based Simulation* Creation of an actual-time virtual model of parking and infrastructure to permit real-time monitoring and forecasting.
2. *Multi-Objective Pareto Optimization*New way of managing the conflict of interest like search time, energy dissipation, and traffic density by Pareto front optimization.
3. MDP-Based Decision-Making: Translating the parking allocation problem into an MDP that can incorporate real-time dynamic decision-making.
4. *PSO for Global Optimization* Interfacing of PSO with the GA so as to improve and enhance the Pareto-dominant solutions identified.
5. *Performance Evaluation and Comparison* Many samples for proving the efficiency of the proposed framework in comparison with the basic methods like Round Robin, Random Allocation, and the Threshold Based methods.

### Paper organization

The rest of the paper is organized as follows: In Section 3 related Work, a comprehensive literature review of smart parking systems, optimization algorithms, and the use of Digital Twin technology is presented. In section 4 system Model and Proposed Framework provides an overview of the studying system model, the formulation of the problem, and the theoretical background of the proposed framework. The section 5 describes Pareto Optimization, an MDP-based decision-making algorithm, and PSO for global optimization algorithms are all described in clear pseudocode in Proposed Algorithms. The section 6 explains the simulation environment and describes the overall experimental setup for the validation of the present framework, the setting of parameters, and the criteria for measurement. Section 7 discusses performance comparisons with prudent baseline methods, trade-off analysis, and results visualizations are discussed in the Results and Discussion section to bring out the efficacy of the proposed framework. Finally, section 8 contains Conclusion and Future Work provides the conclusions and future work to improve the proposed solution for extending the proposed solution.

## Related work

This section presents an overview of the literature with regard to smart parking systems, optimization techniques, and the application of Digital Twin technology in smart systems. This not only underlines the areas where current methodologies fail, for example in scalability, consideration of multiple objectives, and integration.

The high levels of growth in city centers have put serious pressure on parking management hence the innovation of smart parking. Web of Things (WoT) based systems have become common for solving these problems. Sensors are placed in parking slots to check their availability and use IoT to relay information to drivers in real-time^15–17^. For example, a smart parking framework utilizing the IoT is proposed in^18^, which assists clients in finding parking areas through cloud computing and mobile applications. In the same fashion, cloud systems with interfaces to the sensors in parking zones in towns and cities have been found to decrease search times and increase parking spot efficiency^19^. While conventional parking systems are on the other hand based on conventional fixed slot allocation methods or even manual observations, they are not well suited for dynamic parking systems like those in congested urban zones. These systems do not possess the capacity to adjust to current requirements and traffic situations. IoT-based solutions try to address this problem by offering constant updates; however, these approaches mostly improve local solutions rather than the overall system solutions^20^. Further developments introduce the incorporation of what is known as edge computing to minimize latency so as to enhance decision-making capabilities^21^. Smart parking systems II is based on the fact that edge computing works close to where data is being collected hence minimizing transference load. However, the priority of most current systems lies in solving single-object optimizations, for example, search time or parking lot occupancy^22^ and thus do not solve the problem of multi-attribute optimization such as energy, traffic, and utilization.

Optimization is a central factor when it comes to the distribution of parking resources and there are a number of strategies developed and still proposed with the use of heuristic, metaheuristic, and multi-objective. It is structured well to accommodate large and complex searches for which Genetic Algorithms (GA) are well suited. For example, a GA-based smart parking model discussed by the authors in^23^ addresses space allocation based on current traffic flow as well as the choice of users. Like most heuristics, GA has a high computational cost in large systems; hence poorly scaled for large parking systems. Some other methodologies, belonging to the family of Swarm Intelligence Techniques, such as PSO and ACO, have been also used for parking optimization. The use of PSO has been applied to reduce search time and uniformly distribute parking spaces across the zones^24^, while ACO has been applied to enhance vehicle circulation and prevent jamming in dynamic conditions^25^. As these algorithms are highly efficient in real-time optimization, they are sensitive to hyper-parameters that need to be cautiously optimized for convergence^26^. Multi-criteria optimization methods aim at solving conflicting objective functions in urban parking systems that include; time to search for a parking slot, energy used, and traffic jams. Take Pareto Front Optimization has been applied to satisfy the need for a high user satisfaction rate and low energy consumption at the same time while increasing the level of resource utilization dramatically^27,28^. However, these methods target local searches and do not consider the existence of global search heuristics. As remarkable achievements have been made till date, the existing methods of optimization are still faced with isolation and negligence of integration with the predictive model used in tackling the real problem.

Recently, the Digital Twin (DT) has rapidly emerged in smart systems to develop precise replicas of physical objects which can actually facilitate monitoring, analyzing, and even controlling for continuous decision-making about resource management^29^. Smart parking, for instance, sees DT complement real-time sensor information to mimic parking availability and predict future space utilization, for example, a DT-based parking system discussed in^30^, explores several “what-if” cases to adaptively assign parking lots according to new needs. Likewise, a DT-enabled urban traffic management system in^31^ proposed the use of real-time simulations to minimize traffic build-up and enhance traffic patterns. Besides parking, DT has been used in scheduling resources for manufacturing smart^32^ and energy-efficient systems^33^ whereby integrating AI and IoT, DT helps in simulating the future use of infrastructure. However, the improvement of DT in smart parking systems is still low. The majority of the applications only work in the context of a prediction space but they do not provide links to optimization frameworks for online decision making. Also, computational issues are observed as the frameworks intended for modeling extensive urban systems involve the integration of real-time data and have grand computational scales^34^. It is possible that filling these gaps could eventually lead to the optimization of DT in delivering improved and optimized parking prospects.

Despite the overall advancements in the field of smart parking systems the following challenges still exist which constrain the implementation of these systems in the context of large-scale cities. This is an area of concern because IoT-based optimization methods that exist falter when the vehicle density increases^35^. Other problems, Schaffer’s GA and Dorigo’s ACO for instance, encounter computational complicacy difficulties, particularly when applied at large^36^. Time is also an important factor They mostly use cloud computing for computation, which leads to latency problems in real-time functioning. However, while edge computing is suggested, its combination with predictive and optimization models has not been actively implemented^37^. Moreover, most of the time real multi-objective trade-offs in the search space are not evaluated in a general way, most of the optimization models and methodologies merely aim at a single objective such as minimizing the search time or maximizing occupancy^38^. Insufficient work has been dedicated to achieving contradictory objectives including time, energy usage, and traffic^39^. More specifically, there are integration gaps, in which current work does not link Digital Twin Technology, optimization algorithms, and decision-making paradigms like the Markov Decision Process (MDP). The lack of a blended and integrated design leads to inferior performance in dynamic and challenging small city conditions^40,41^.

Recently, there have been many researches in vehicular technology and smart mobility that explore some aspects of intelligent transportation systems, but the smart parking solutions have not been optimized well. In his work^42^, Yao et al. have introduced an automotive radar optimization for V2I communication in spectrally crowded environments for vehicular sensing and not coupled with a real-time parking optimization. Similarly, An et al.^43^ also proposed the cascade attention-modulation fusion algorithm to improve autonomous vehicle navigation significantly but did not address the real-time dynamic parking space allocation. In particular, Ma et al.^44^ studied energy conservation and emission reduction in urban waste control, proposing the policies are effective in terms of sustainability but have not considered the effect of the energy-efficient smart parking systems. Fog based distributed routing strategy has been developed by Sun et al.^45^ for V2V communication which optimized vehicular networking but ignored the application in Dynamic Parking Space Management. However, the state of predictive modeling for routing as Sun et al.^46^ apply to V2V networks does not extend to real-time smart parking optimization. Similarly, Sun et al.^47^ add trajectory-based routing to improve message delivery in urban VANETs, but this route optimization does not take into account a multi-objective optimization to allocate the parking resource. For example, Wang et al.^48^ proposed the use of a multisensor-based measurement quality control system for vehicle navigation, although it is not associated with smart parking space optimization. A disparity segmentation-based vehicle detection was proposed by Li et al.^49^, which improves the capability to track the vehicle though without an emphasis on the efficient parking allocation mechanisms. In the work of Zhou et al.^50^, a Gaussian risk model for vehicle trajectory planning in the scene of driving safety is given, which, however, does not take onboard active real-time parking slot management. Li et al.^51^ study the positioning precision of LiDAR-OpenStreetMap matching, a very useful operation for autonomous navigation, but does not optimize urban parking allotment. As shown in Liu et al.^43^, information security and freshness of vehicular data sensing via the blockchain improve but do not incorporate multi-object optimization for smart parking. While powerful, Liang et al.^52^ devise a deep reinforcement learning framework for high-speed autonomous driving that has not yet been used to make real-time parking decisions. Traffic management by optimizing road efficiency was the focus of curvilinear multilane merging and platooning for Wu et al.^53^, whereas urban parking solutions were not addressed. Xiao et al.^54^ proceeded with occlusion-aware information sharing for autonomous vehicles, which improved perception but has no direct application in smart parking allocation. Based on these studies, several research gaps persist: the absence of real-time multi-objective optimization techniques that consider search time, energy efficiency, and congestion reduction; the lack of integration of Digital Twin Technology, Pareto Optimization, and MDP for dynamic decision-making in parking systems; limited adoption of hybrid AI-based approaches that combine reinforcement learning with traditional optimization models^55,56^; and an under-explored application of blockchain for secure, adaptive parking slot management. These gaps are our justification for the integration of the Digital Twin Technology, Pareto Front Optimization, Markov Decision Process (MDP), and Particle Swarm Optimization (PSO) into the smart parking system so as to boost the performance of smart parking system in an urban environment [].

One of Rong et al.^57^’s work is Du-bus, which is a real-time bus waiting time estimation system based on multi-source data fusion. The improvements they bring to public transportation do not substantially apply to dynamic parking resource allocation, which is important for optimizing urban mobility. Li et al.^58^ also proposed an integrated decision-making and motion-planning framework to enhance the driving stability of autonomous vehicles and decrease autonomous vehicle oscillations. However, this work provides trajectory planning while it does not include the multivariate optimization of urban parking management.

**Fig. 1:**
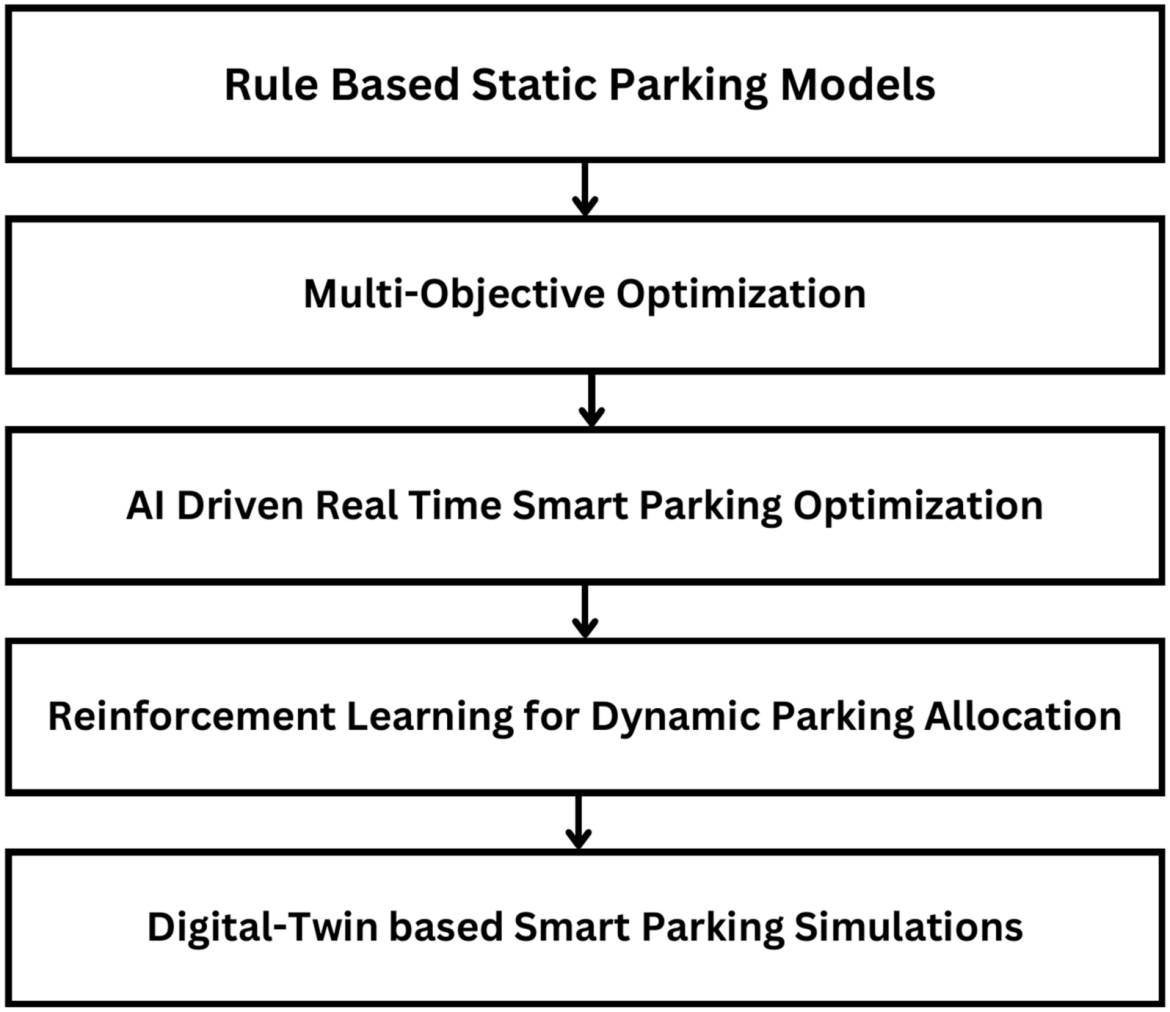
Research progression in smart parking optimization.

Recently, Peng et al.^59^ studied task offloading in the Internet of Autonomous Vehicles (IoAV) with extreme weather conditions by means of reinforcement learning-based dynamic pricing. The computation offloading scheme is optimized but does not take parking space allocation into consideration in autonomous vehicular networks. However, Ma et al.^60^ have studied large-scale health intervention programs and how they impact healthcare expenditures but only from the socio-economic side and do not present technical solutions for smart mobility optimization. Song et al.^61^ designed a spatiotemporal cognitive risk distribution model for a subjective driving risk prediction model that improves road safety. However, their approach addresses risk assessment but does not relate to real-time decision-making frameworks for safe parking. Visual feature fusing for heterogeneous traffic flow prediction was optimized to improve traffic congestion forecasting by Wang et al.^62^. Nevertheless, they do not carry predictive modeling over to parking management, where congestion is a key issue. Li et al.^63^ introduce a degradation twin model for rolling bearing performance application with the model assumption that the faults evolve in parallel. This research does not investigate digital twin applications in parking space optimization, although this is relevant for predictive maintenance. As energy energy-aware low latency routing model for mobile edge computing, EALLR is proposed by Zhang et al.^64^. While their model strengthens data-driven energy-efficient routing, it does not have direct applications to solve the problem of energy-aware parking slot allocation. Rotational LiDAR data is used in the work of Chen et al.^65^ to jointly estimate scene flow and moving object segmentation. The contribution of this research is a new real-time perception in urban environments, yet its integration with smart parking monitoring and allocation is not pursued. Lin et al.^66^ investigated historical occurrences of urban sound in terms of time and their evolution. Yet, this work does not offer technical solutions to adaptive parking systems, which are seen in modern-day cities. Resources distribution in air-ground integrated mobility is optimized with personalized distribution based on the channel knowledge map in^67^. This work provides an improvement to resource allocation in connected transportation systems, but this does not directly address real-time parking management and congestion control. Figure 1 shows the progression of research in Smart Parking Optimization based on Rule-based, Static and Multi-Target Optimization based on Digital Twin, Reinforcement Learning, Pareto and PSO. The table 1 provides a comparative analysis of smart transportation and optimization studies that have taken place recently, focusing on the contribution made by each study, as well as its approach to optimization, the application domain, and the limitations to position the proposed work as a holistic and alternative solution to solve real-time, scalable and energy-optimized smart parking management.

**Table 1:** Comparative analysis of recent studies in smart transportation and optimization.

Study	Key contribution	Optimization approach	Application domain	Limitations
Yao et al.^42^	Automotive radar optimization for V2I communication	Spectrum-aware radar optimization	Connected vehicles	No consideration for parking resource allocation
An et al.^43^	Traffic sign recognition using cascade attention-modulation fusion	Deep learning-based image recognition	Autonomous driving	Lacks integration with real-time parking systems
Ma et al.^44^	Impact of emission reduction policies on urban waste control	Policy-driven sustainability modeling	Urban environmental management	No direct link to mobility optimization
Sun et al.^45^	Fog-based distributed routing for V2V communication	Decentralized edge computing for vehicle networks	Smart transportation	Does not address parking resource optimization
Sun et al.^46^	VANET-based routing using ARIMA model	Predictive traffic modeling	Vehicular ad-hoc networks	No focus on parking space prediction
Wang et al.^48^	Multi-sensor fusion for improved vehicle navigation	Sensor-based localization enhancement	Autonomous vehicles	Lacks application in dynamic parking allocation
Li et al.^49^	Disparity segmentation-based vehicle detection	Image processing for object detection	Smart surveillance	Does not integrate parking space monitoring
Zhou et al.^50^	Gaussian risk model for vehicle trajectory planning	Probabilistic modeling for motion prediction	Autonomous vehicle control	No application to parking space optimization
Li et al.^51^	LiDAR-OpenStreetMap matching for vehicle positioning	Hybrid LiDAR and mapping-based localization	Smart navigation systems	Does not consider real-time parking allocation
Liu et al.^68^	Blockchain-enabled vehicular data sensing	Secure data transmission via blockchain	Intelligent transportation	Lacks integration with parking resource allocation
Liang et al.^52^	Deep reinforcement learning for high-speed cruising performance	AI-based motion control	Autonomous vehicles	No parking resource optimization component
Wu et al.^53^	Multi-lane merging and platooning control in curved roads	Traffic flow optimization	Highway transportation	No dynamic parking management integration
Xiao et al.^54^	Perception task-oriented information sharing in CAVs	Sensor fusion for occlusion-free vision	Connected autonomous vehicles	No focus on smart parking optimization
Rong et al.^57^	Real-time bus waiting time estimation using multi-source data	Data fusion for public transport scheduling	Smart public transit	No adaptation for parking resource optimization
Li et al.^58^	Decision-making and motion planning for oscillation-free driving	AI-based motion prediction	Autonomous vehicle control	No link to smart parking decisions
Peng et al.^59^	Task offloading for IoAV under extreme weather conditions	Reinforcement learning-driven task allocation	Internet of Autonomous Vehicles (IoAV)	Lacks application in parking space scheduling
Wang et al.^62^	Visual feature fusion for heterogeneous traffic prediction	AI-based traffic modeling	Traffic flow optimization	No extension to parking congestion management
Zhang et al.^64^	Energy-aware low-latency routing in mobile edge computing	Adaptive routing in vehicular networks	Edge-based transportation systems	No implementation for energy-efficient parking
Yue et al.^67^	Resource allocation in air-ground mobility networks	Channel knowledge map-based resource distribution	UAV-assisted mobility	Does not address parking management in urban environments
**Proposed Work**	Multi-objective optimization for smart parking using DT, MDP, and PSO	Hybrid AI + heuristic optimization	Smart parking	Addresses real-time parking allocation, scalability, and energy efficiency

## System model and proposed framework

**Fig. 2:**
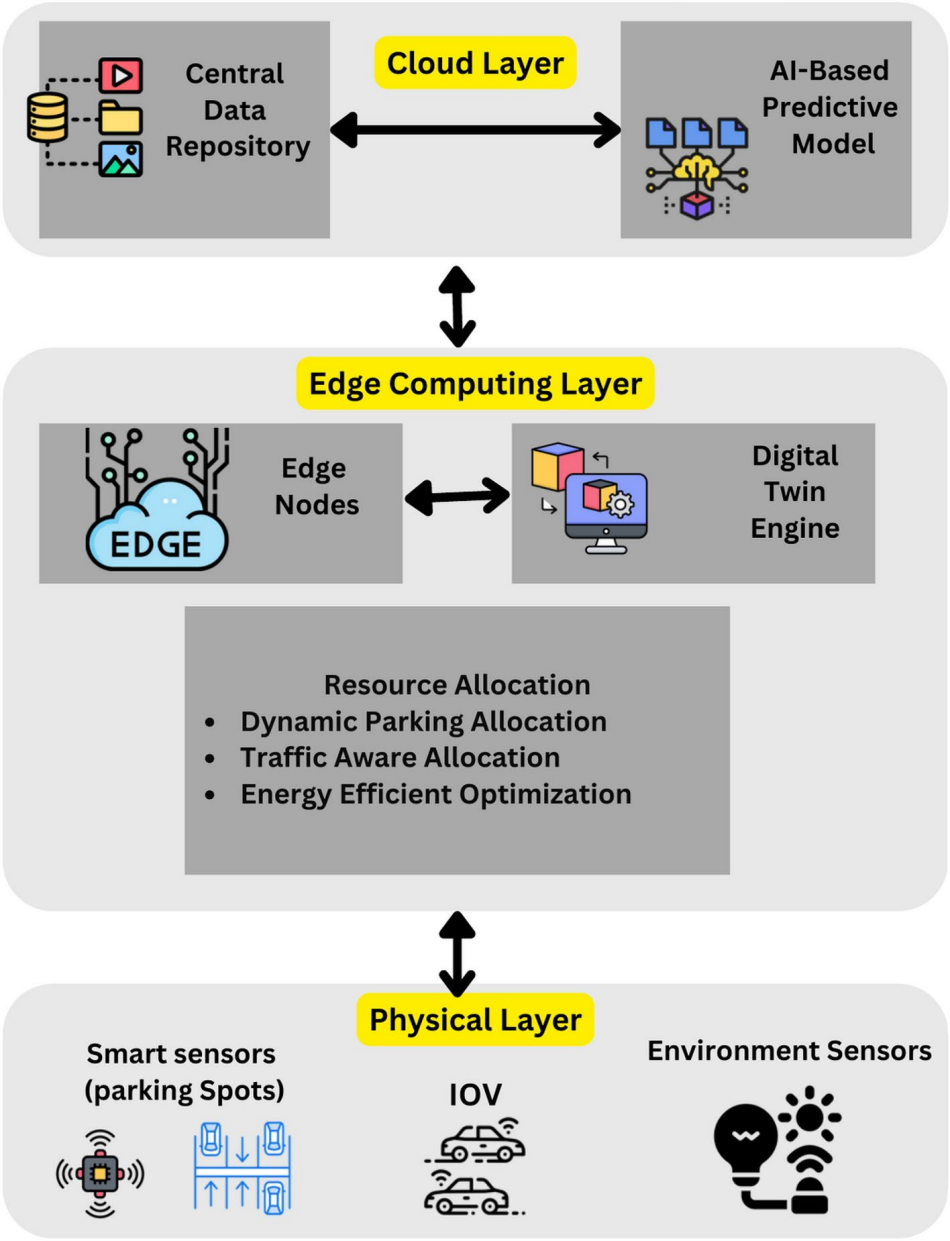
System model for smart parking solutions.

Current real-world parking optimization methods are mostly heuristic or employ single-objective optimization approaches that can barely solve volatile and heterogeneous real-life problems. The proposed framework is a new multi-objective method that addresses the challenged problem of parking space allocation and is based on combining Digital Twin Technology, Pareto Front Optimization, Markov Decision Process (MDP), and Particle Swarm Optimization (PSO). Currently, the proposed framework does not rely on static methods or outdated heuristics but employs fast predictive modeling (DT) and real-time optimization methods like Pareto Front, Multi-Decision Process (MDP), and Particle Swarm Optimization (PSO) for efficient parking allocation. This translates into an efficient, scalable, and energy-effective smart parking solution that is superior to the conventional approaches. The system model for “Smart Parking Solutions” is presented in Fig. 2. this paper presents state-of-the-art technologies and is divided into three main layers to provide real-time management and computational analysis as well as efficient allocation of parking resources. At the heart of the architecture is the use of a hierarchical approach that facilitates the flow of data as well as decision-making as well as the management of resources.

At the Physical Layer, the system and the infrastructure, and the parking vehicles are engaged through physical means through smart sensing and monitoring interfaces. Some of the IoT smart parking systems are located at parking areas to monitor occupancy status and times of entry and exit, parking space, and parking time. Similarly, connected vehicles in the IoV system are involved in active dialogue with the parking facilities and convey data, including vehicle type, parking time estimate, and user preferences. Moreover, environment probes like the roadside units or cameras or LiDaR undertake the task of recording traffic flow, congestion around parking areas or even performing plaque number recognition or space detection. These components allow the proper capture of other data relevant to the system’s functions.

The Edge Computing Layer is the layer where all the processing of data in real-time, collecting data, making decisions, and optimizing processes occur. Proposed as edge nodes, data from parking facilities or nearby sites are first computed at those facilities to minimize latency while offering immediate parking solutions. This layer integrates a Digital Twin Engine that generates a duplicate model of the parking structure which predicts parking conditions including the number of vehicles entering or exiting the facility, available spots, and traffic intensity. Within this layer, resource allocation algorithms have been included for making better decisions. These include the Dynamic Parking Allocation Algorithm which provides available parking spaces and user preference; the Traffic-Aware Allocation Algorithm, which redistributes traffic flows; and the Energy-Efficient Optimization Algorithm, which decreases idle search and excessive fuel consumption improving energy uses.

The Cloud Layer functions as an instrument for the efficient storage and management of records and data in the long term, as well as an exceptional tool for a detailed analysis of a business and its systems’ dependability. Long-term parking usage data of the parking space, traffic of vehicles and congestion patterns are stored in the centralized database as it is the source of historical data in all the layers in a network in case of failure of the edge nodes. Also, the application of innovative machine learning algorithms offers future parked car demand and tendencies’ predictions. These models are constantly updated and in sync with edge nodes such that real-time decisions are in turn influenced by historical reference, trends and forecasts.

By integrating the functionalities of the Physical Layer, Edge Computing Layer, and Cloud Layer, the system forms a stable and optimum digital twin-aided edge computing architecture for smart parking applications capable of adaptive resource management, avoiding traffic jams, and energy conservation.

### Data flow and communication

The flow of data and communication in a digital twin-assisted smart parking system is organized and highly optimized for a better working system. It starts with data gathering, and IoT sensors, installed in parking places, identify them as vacant and send occupancy information to the nearest edge nodes. Concurrently, vehicles broadcast their coordinates parking profile, and other related metadata, whereas environmental sensors and cameras detect the traffic and environmental signals. In the next step, data is computed at the edge nodes themselves to produce real-time information. The digital twin replicates the parking lot, estimates short-term parking lot availability and traffic, and resource allocation algorithms determine parking facility assignments. Once optimized and implemented, the system informs users through the mobile apps or the in-car infotainment system to proceed to the designated parking spot that the system has assigned to him/her based on an optimized navigation system. Another closed loop follows for constant and efficient enhancement of the solution by sending data about parking availability and customer satisfaction to edge nodes and the cloud layer.

The digital twin improves the system through real-time insight into parking lot occupancy and traffic flow as well as behaviors of the vehicles. It is used to predict future parking requirements using both current and past data, with various allocations to yield the best solution. The twin also allows dynamic updates with reference to real-world changes on the physical layer. Algorithms for distributing resources strengthen the system’s performance even more. Dynamic parking allocation algorithm provides efficient ways for space capacity and allocation depending on availability, vehicle location, and user’s choice based on fast decisions like a greedy algorithm or graph approaches. The traffic-aware allocation algorithm reallocates autonomous vehicles to regions with low traffic density based on traffic congestion and the number of vehicles in the region. It also uses reinforcement learning to update policies. The derived optimal Parking Search Control strategy gives low fuel and energy consumption by applying a cost-time-energy multi-objective optimization. The Digital Twin (DT) has three layers, namely the Physical Layer which gathers information from parking availability and congestion through IoT sensors, the Digital Layer accommodates a virtual copy of the actual physical parking lot, and the Decision Layer which uses artificial intelligence algorithms for parking resources scheduling. Real-time updates, the DT system draws from past occurrences and makes new forecasts on the amount of time it takes to search for one or find the right parking space to allocate.

The efficiency of the system that has been proposed is measured based on certain parameters. The effectiveness of parking spaces is determined through the use of utilization rate as it shows how many of the set parking spaces are in use while search time is used to determine the number of times a driver spends in an effort to locate an available parking space. Traffic congestion reduction measures the extent to which the amount of car movements is increased around parking zones, energy efficiency measures the extent to which the amount of fuel or energy used is minimized and lastly, user satisfaction is a measure of the extent to which drivers found the system easy to use and dependable. This holistic approach then guarantees an integrated, effective, and user-centered smart parking system. Due to trade-offs between multiple objectives, our framework utilizes Pareto Front for search time minimization, energy efficiency, traffic congestion, and fairness of parking slot allocation. A personalized parking strategy is used in order to select the best among Pareto-optimal solutions based on the current situation. The real-time parking allocation is formulated as a sequential decision-making problem in the MDP-based decision-making framework as illustrated by the following; The State Space encompasses parking status, incoming arrival rate, and congestion level. Meanwhile, Action Space encompasses parking slot assignment, rerouting or indeed the delaying of a vehicle entry into a certain parking. The Reward Function aims at minimizing waiting time and penalty due to congestion and at the same time, maximizing free space availability while the Transition Model is probabilistic and is derived based on the prior probabilities or likelihood of parking allocation. Utilizing a specific method of reinforcement learning called Q-learning, the MDP model allows for daily adjustments to the time and space of parking according to demand, making for better efficiency. Table 2 contains all the symbols and their description used in this research.

**Table 2:** Symbols and their descriptions used in the Smart Parking Allocation Framework.

Symbol	Description
*N*	Number of parking spaces.
*M*	Number of vehicles requesting parking.
*K*	Number of traffic zones.
*P*	Set of parking space availability states, $P = \{P_i \mid i = 1 \text { to } N\}$.
*L*	Set of traffic congestion levels for each zone, $L = \{L_k \mid k = 1 \text { to } K\}$.
*R*	Set of vehicle parking requests, $R = \{R_j \mid j = 1 \text { to } M\}$.
$T_s$	Search time for vehicles to find a parking space.
$E_s$	Energy consumption of vehicles while searching for parking.
*C*	Traffic congestion level in a specific zone.
*U*	Space utilization, indicating the proportion of utilized parking spaces.
$w_1, w_2, w_3, w_4$	Weights for the multi-objective optimization function.
*X*	Initial population of solutions in Pareto optimization.
$X^*$	Pareto-optimal set of solutions.
*Q*(*S*, *A*)	Q-value for a state-action pair in the MDP.
*S*	Current state, including parking availability, congestion levels, and requests.
*A*	Action taken, such as assigning a vehicle to a parking space.
*R*	Reward function in the MDP.
$\gamma$	Discount factor for future rewards in the MDP.
$v_{ij}$	Velocity of a particle in PSO.
$x_{ij}$	Position of a particle in PSO, representing parking assignments.
$\omega$	Inertia weight in PSO.
$c_1, c_2$	Acceleration coefficients in PSO for personal and global best updates.
$r_1, r_2$	Random numbers between 0 and 1 used in PSO updates.
$p_{\text {best}}$	Personal best position of a particle in PSO.
$g_{\text {best}}$	Global best position among all particles in PSO.

## Mathematical modeling of proposed system

The primary objective is to minimize parking search time ($T_s$), energy consumption ($E_s$), and traffic congestion (*C*), while maximizing parking space utilization (*U*). The objective function is expressed as:

1 $$\begin{aligned} \text {Minimize } F = w_1 T_s + w_2 E_s + w_3 C + w_4 (1 - U) \end{aligned}$$

Where $T_s$ represents the average parking search time, $E_s$ denotes the energy consumption during parking searches, and *C* reflects the traffic congestion level. Additionally, *U* indicates the parking space utilization ratio, while $w_i$ are the weight coefficients assigned to prioritize the respective terms based on the objectives of the optimization problem.

The system variables are defined as follows: *N* represents the total number of parking spaces, while *M* denotes the total number of vehicles requesting parking. The availability of parking space *i* is indicated by $P_i$, where $P_i = 1$ if the space is available and $P_i = 0$ otherwise. The distance between vehicle *j* and parking space *i* is given by $D_{ij}$, and the energy consumption for vehicle *j* to reach parking space *i* is represented by $E_{ij}$. Additionally, $L_k$ measures the traffic congestion level in zone *k*, and $U = \frac{\text {Number of occupied spaces}}{N}$ defines the utilization ratio, which indicates how effectively the parking spaces are being utilized. Parking assignment Constraint in which each vehicle can only be assigned to one parking space:

2 $$\begin{aligned} \sum _{i=1}^N x_{ij} = 1, \quad \forall j \in \{1, 2, \dots , M\} \end{aligned}$$

Availability Constraint considers that a parking space cannot be assigned to more than one vehicle:

3 $$\begin{aligned} \sum _{j=1}^M x_{ij} \le P_i, \quad \forall i \in \{1, 2, \dots , N\} \end{aligned}$$

Congestion Threshold constraint means traffic congestion in a zone must not exceed the predefined limit $L_{\text {max}}$:

4 $$\begin{aligned} L_k \le L_{\text {max}}, \quad \forall k \in \{1, 2, \dots , K\} \end{aligned}$$

The objective function after rewriting is as follows:

5 $$\begin{aligned} F = \sum _{j=1}^M \sum _{i=1}^N \left( \alpha T_{ij} x_{ij} + \beta E_{ij} x_{ij} \right) + \gamma \sum _{k=1}^K L_k + \delta \left( 1 - \frac{\sum _{i=1}^N \sum _{j=1}^M x_{ij}}{N} \right) \end{aligned}$$

where:

6 $$\begin{aligned} T_{ij} = \frac{D_{ij}}{v_j}, \quad E_{ij} \propto D_{ij} \end{aligned}$$

Here, $T_{ij}$ is the time taken by vehicle *j* to reach parking space *i*, and $v_j$ is the speed of vehicle *j*. $E_{ij}$ is the energy consumption proportional to distance $D_{ij}$.The objective incorporates the actual parking measurements as well as the global limitations to balance and optimize the parking distribution.

The Digital Twin predicts system behavior using real-time and historical data. To evaluate congestion levels, the Digital Twin estimates the density of vehicles in each zone:

7 $$\begin{aligned} P_d(i, t) = f(\text {historical trends, current requests, external factors}) \end{aligned}$$

Simulated traffic congestion can be tracked by following formulae:

8 $$\begin{aligned} L_k(t) = \frac{\sum _{j \in \text {zone } k} x_{ij}}{\text {zone area}} \end{aligned}$$

This model offers flexibility for parking allocation in relation to demand and traffic density by offering forecasts in advance.

The average search time evaluates the efficiency of the system in reducing delays for users:

9 $$\begin{aligned} T_s = \frac{\sum _{j=1}^M \sum _{i=1}^N T_{ij} x_{ij}}{M} \end{aligned}$$

This metric computes the time spent by all vehicles searching for parking spaces and averages it over the total number of vehicles.

The energy consumption measures the system’s efficiency in minimizing fuel usage or battery drain:

10 $$\begin{aligned} E_s = \frac{\sum _{j=1}^M \sum _{i=1}^N E_{ij} x_{ij}}{M} \end{aligned}$$

This metric aggregates the energy consumed by all vehicles during the parking search and calculates the per-vehicle average.

Traffic congestion evaluates the system’s effectiveness in managing vehicle density in various zones:

11 $$\begin{aligned} C = \sum _{k=1}^K L_k \end{aligned}$$

Congestion is calculated as the sum of vehicle densities across all zones, highlighting areas that need better traffic management.

Space utilization assesses how efficiently the available parking spaces are used:

12 $$\begin{aligned} U = \frac{\sum _{i=1}^N \sum _{j=1}^M x_{ij}}{N} \end{aligned}$$

This metric computes the proportion of occupied parking spaces, indicating how well the system maximizes resource utilization.

In multi-objective optimization when using the Pareto front, some of the major goals are reduced search time ($T_s$), optimizing customers’ interactions to enhance their productivity and avoid wastage of power. Sustainability is addressed by minimizing energy consumption ($E_s$), while traffic congestion (*C*) is decreased to improve urban transportation. Additionally, increasing the density of space utilization (*U*) ensures efficient use of resources. It is therefore necessary for managers to carefully evaluate the various resources required in an organization to facilitate optimum utilization. The Pareto front defines a set of optimal compromises between these objectives and aids decision-makers in comparing different solutions. A scalarized solution incorporates all objectives in terms of weighted coefficients, denoted as:

13 $$\begin{aligned} F = w_1 T_s + w_2 E_s + w_3 C - w_4 U, \end{aligned}$$

where $w_i$ are the weights that, as mentioned before, may be adjusted dynamically and correspond to system priorities. This approach allows for achieving one goal whilst at the same time addressing another conflicting goal, and it also enables the adjustment of the solution based on real-time data for parking optimization.

Dynamic optimization of the parking resource allocation can be solved using the reinforcement learning technique based on Markov Decision Processes (MDP). $S$ is the state space that synchronizes the current system environment including available parking spaces ($P_i$), traffic densities in various zones ($L_k$), and parking requests ($R_j$). Actions ($A$) are the allocation of parking lots to cars, where action $x_{ij}$ is 0 for a car not being assigned to a particular lot or 1 otherwise. The reward function ($R$) assigns a fitting measure of the optimality of an action to achieve multiple objectives, including minimizing search time ($T_s$), energy usage ($E_s$), traffic congestion ($C$), and to maximize communal space utilization ($U$). The reward function is defined as $R = -(w_1 T_s + w_2 E_s + w_3 C - w_4 U)$, whereby $w_i$ are scaling factors that determine the preference for the respective objective. For solving the parking allocation policy ($\pi ^*$), where the goal is to maximize the benefit, those techniques include Q-learning or Deep Reinforcement Learning (DRL).

14 $$\begin{aligned} \pi ^* = \arg \max _\pi \mathbb {E} \left[ \sum _{t=0}^\infty \gamma ^t R_t \right] \end{aligned}$$

These methods refine the decision-making process, step by step, in terms of the expected accumulated reward over time, and selectively change the parking system corresponding to the variation in parking demand and system conditions.

The PSO algorithm is one of the better-known meta-heuristic strategies for optimizing the assignment of vehicles to parking spaces in smart parking systems. Each particle in the swarm represents a potential solution, as a vector of pair-wise assignments of vehicles to parking spaces where each element of the vector is a binary value $x_{ij} \in \{0, 1\}, i \in \{1, \dots , N\}, j \in \{1, \dots , M\}$. The algorithm evaluates the quality of each particle using a scalarized fitness function $F$ that combines multiple objectives. Particles’ positions and velocities are calculated to search the solution space iteratively. The velocity of each particle, $v_{ij}(t+1)$, is influenced by its previous velocity, its own best-known position ($p_{ij}$), and the global best-known position ($g_{ij}$), calculated as:

15 $$\begin{aligned} v_{ij}(t+1) = \omega v_{ij}(t) + c_1 r_1 (p_{ij} - x_{ij}) + c_2 r_2 (g_{ij} - x_{ij}) \end{aligned}$$

where $\omega$ is the inertia weight, $c_1$ and $c_2$ are acceleration coefficients, and $r_1$ and $r_2$ are random numbers between 0 and 1. The position of each particle is then updated as:

16 $$\begin{aligned} x_{ij}(t+1) = x_{ij}(t) + v_{ij}(t+1) \end{aligned}$$

Through these recurrent modifications, particles approach a solution that offers the least search time, energy consumption, and congestion while ensuring maximum space utilization. The PSO algorithm is a scalable and near-optimal solution to the continuous parking assignment problem.

## Proposed algorithms for smart parking allocation framework

**Fig. 3:**
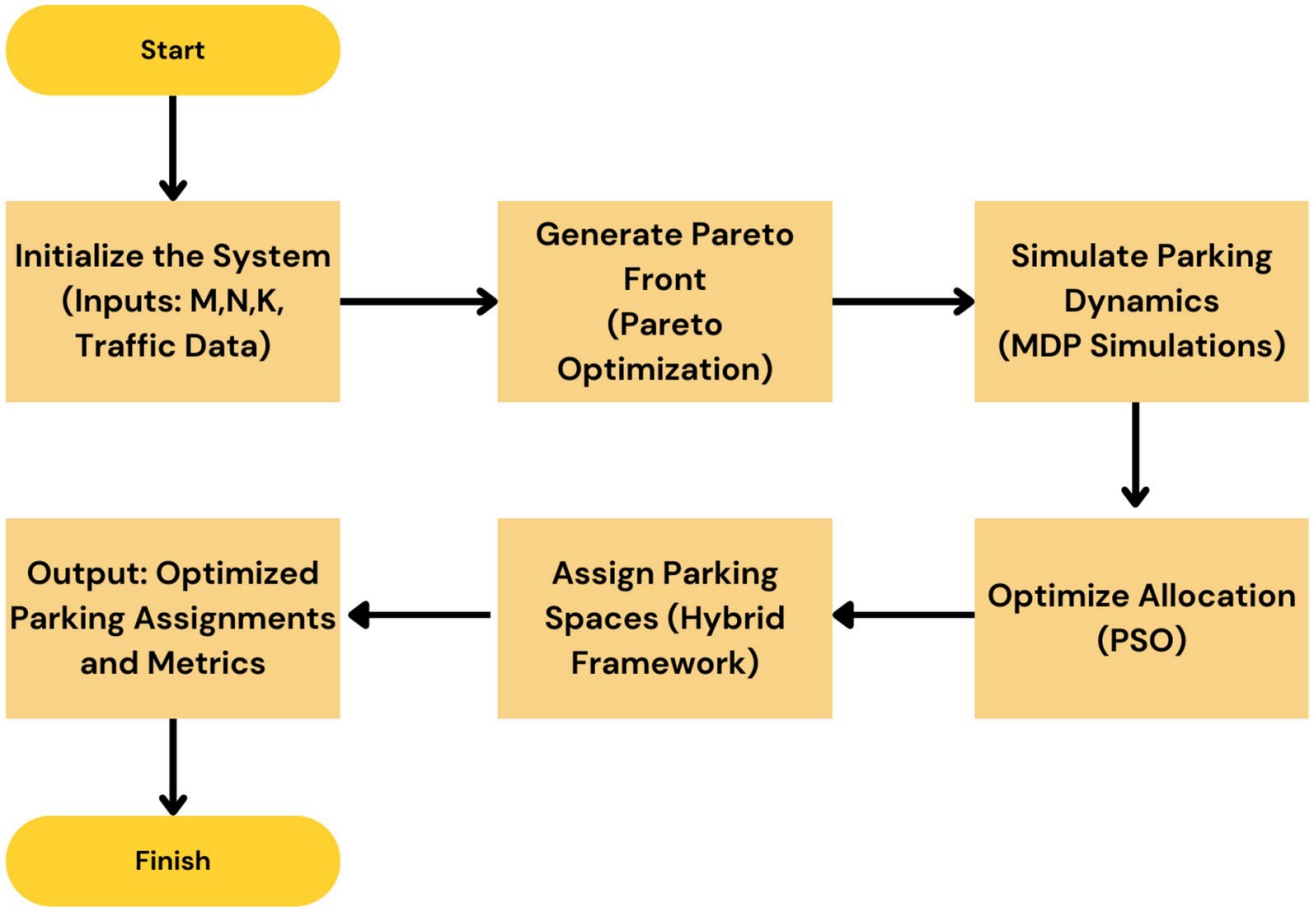
Flow chart of proposed algorithm.

In real-time, the five algorithms combine to allocate parking spaces and manage parking appropriately. The process starts from the Initialization Algorithm 1, which defines the system characterizing factors such as the available parking lot number and traffic intensity level. Second, the Pareto Optimization Algorithm 2 compares and compromises numerous solutions of several objectives such as search time, energy and network congestion and provides a Pareto-optimal front. These solutions are then fine-tuned using the MDP Simulation Algorithm 3, where parking dynamics is assumed to be a sequential decision making problem to maximize expected cumulative reward in the state-action space. The fine tuned solutions are optimally matched worldwide with the help of step:Particle Swarm Optimization Algorithm 4 so that they can finalize the exact distribution plan. Last but not the least the Hybrid Smart Parking Allocation Algorithm 5 uses the output of all the components and provides the parking space to the vehicle and at the same time undertakes the availability and traffic information to make real-life dynamic changes. Such an approach guarantees scaling, optimization, and stability especially in the context of large metropolitan territories.The flowchart of the proposed algorithm is given in Fig. 3.

**Algorithm 1:**
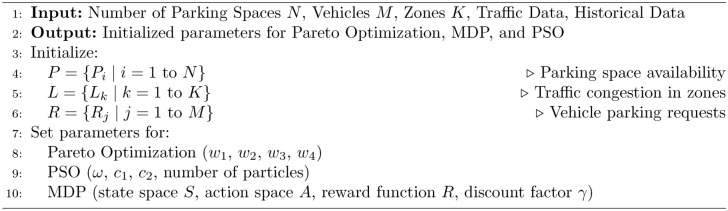
Initialize System

**Algorithm 2:**
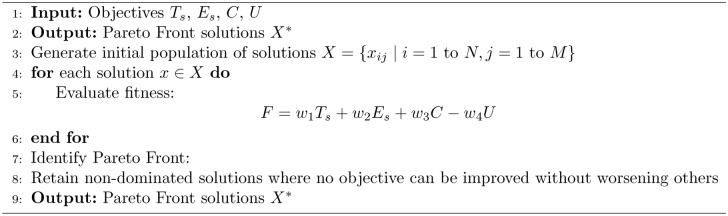
Pareto Optimization

**Algorithm 3:**
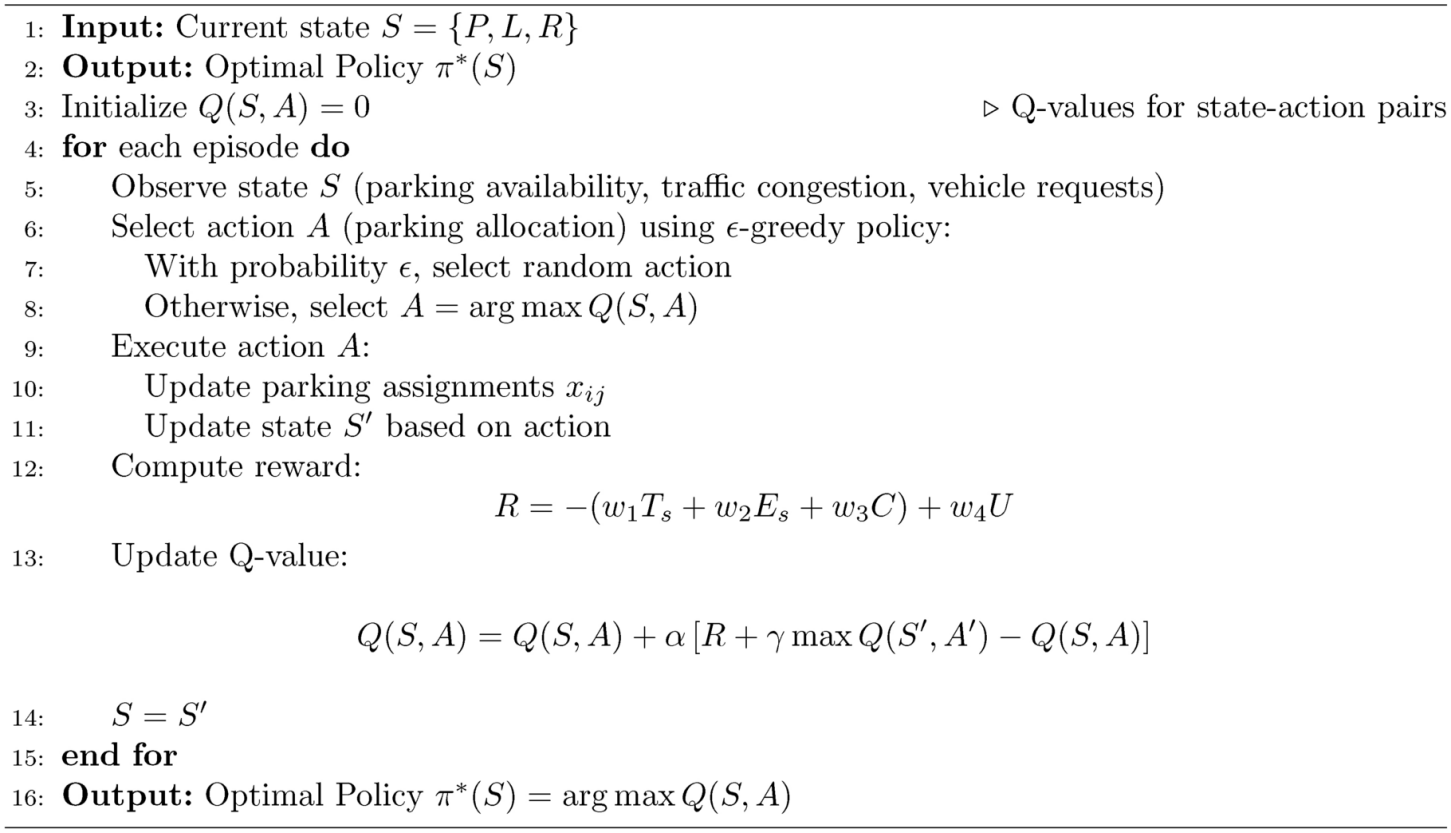
MDP Simulation

**Algorithm 4:**
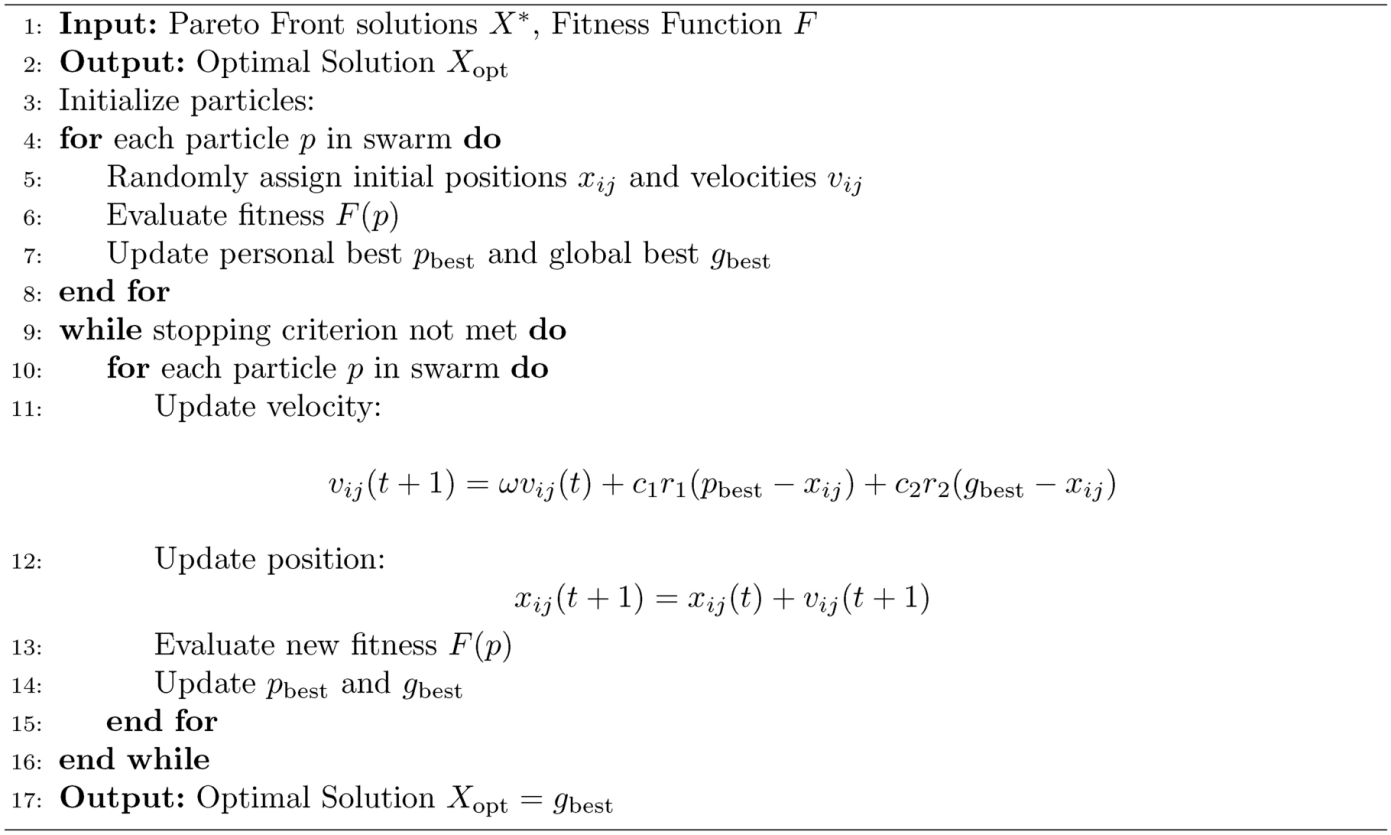
Particle Swarm Optimization (PSO)

**Algorithm 5:**
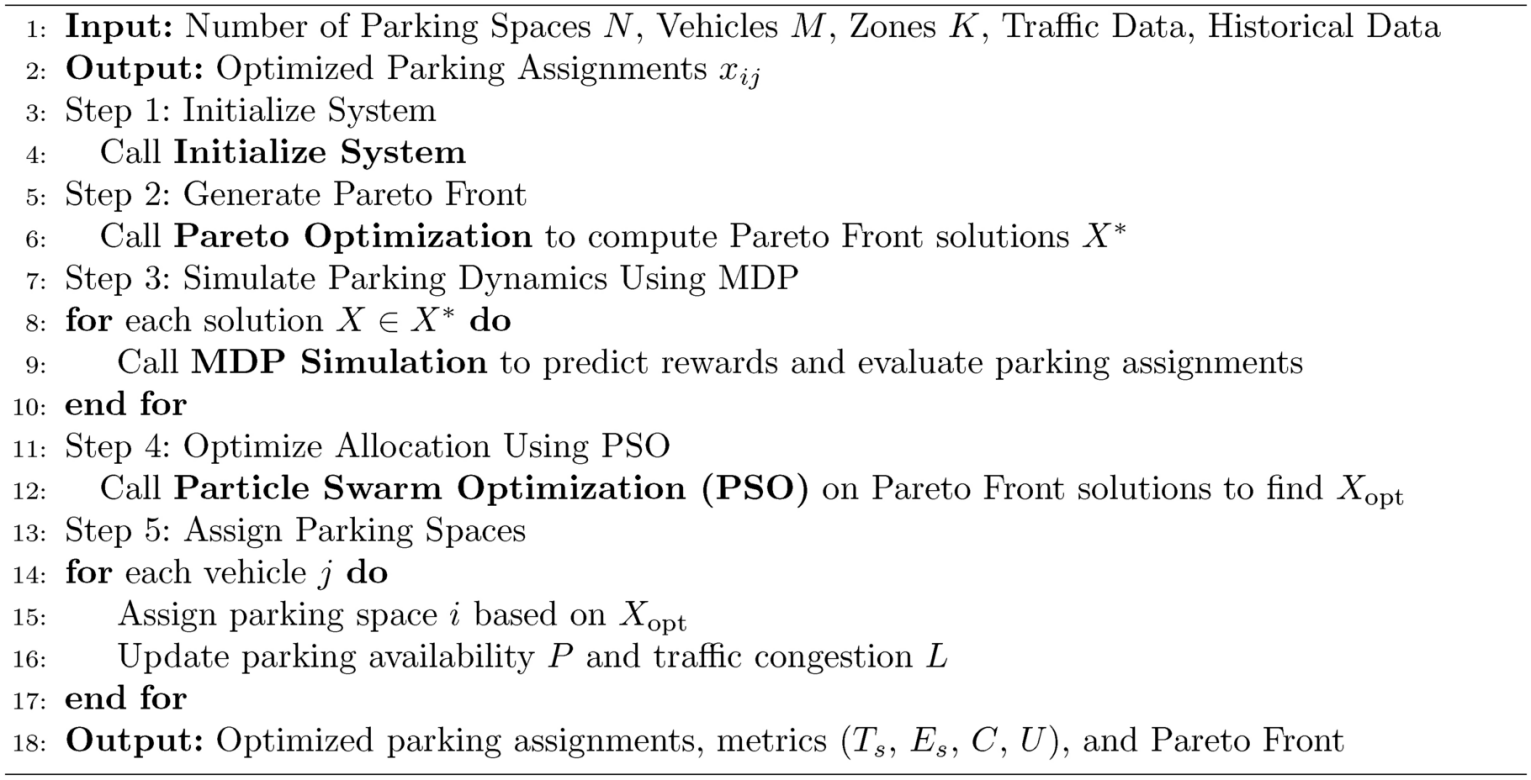
Smart Parking Allocation Framework

## Experimental setup

The provided task is implemented as a smart parking scenario with a Digital Twin model approximating realistic parking conditions. The crowded zones and parking lots along with the placement of vehicles are represented through the digital twin with help from real-time sources like availability and traffic conditions. For local data capturing and real-time data analysis, edge computing nodes are used to make decisions at faster rates. These nodes enable efficient interaction between physical and digital networks, thus operating in real-time.

The simulations were carried out on a real application for urban parking and the traffic density and parking demand were varied. A digital twin was implemented to build a virtual model of the parking system; this model was used for monitoring and prognostic control. The simulation environment was Python-based along with the integration of SUMO (Simulation of Urban Mobility) on a machine regarded as an Intel Core i7 processor, 16 GB RAM, and 1 TB SSD. Python has been used for the simulation and optimization framework since the language offers rich support in numerical calculations, artificial intelligence optimization algorithms, and data handling. TensorFlow and OpenAI Gym support the reinforcement learning module and help in achieving MDP-based decision-making for parking space allocation.

Several baseline algorithms are used and compared to the performance of the proposed framework to test their effectiveness. Round Robin is a kind of scheduling technique, that provides the parking spaces one by one in sequential order without taking into account the traffic conditions or any changes in the parking system environment, this method is less complex and systematic. On the other hand, Random Allocation simply assigns parking spaces according to no particular order; it is designed to be a decree of order by which to compare with other more structured approaches. By identifying quantity thresholds, the Threshold-Based Allocation algorithm awards parking spaces by predetermined distance or zones which are effective, although rigid in comparison to the dynamic outcomes of the algorithms. Moreover, the GA employs evolutionary optimization in a search space wherein the objectives are otherwise in conflict such as search time and space to make it more comprehensive and adaptive to be used as a reference.

When applying the proposed framework, particular parameters are vital for the optimization methods to operate efficiently. Markov Decision Process (MDP) is used for decision making based on system state action and rewards. The PSO set out to optimize the solution space for parking allocations while the Pareto Optimization works to reach the KJV, which balances conflicting objectives. The associated parameters of these algorithms specified in Table 3.

**Table 3:** Parameter Settings for the Optimization Framework.

Parameter	Value
**Markov Decision Process (MDP)**	
State Space (*S*)	Parking availability, congestion, and vehicle requests
Action Space (*A*)	Assigns vehicles to parking slots
Reward Function (*R*)	Optimizes search time, energy, congestion, and utilization
Discount Factor ($\gamma$)	0.9
Learning Rate ($\alpha$)	0.1
**Particle Swarm Optimization (PSO)**	
Number of Particles	50
Inertia Weight ($\omega$)	0.5
Acceleration Coefficients ($c_1$, $c_2$)	1.5 each
Maximum Iterations	100
**Pareto Optimization**	
Objective Weights ($w_1$, $w_2$, $w_3$, $w_4$)	0.3, 0.3, 0.2, 0.2
Population Size	100
Mutation Rate	0.05

The efficiency and effectiveness of the framework and baseline algorithms are measured with different parameters that highlights various aspects of performance. Turnover is calculated through turnover rate and search time, the amount of time it takes for vehicles to find a parking space dependent on the user convenience. Energy consumption evaluates the fuel or energy on the use during the search process and one with the label of sustainability. Traffic congestion measures the degree of congestion of parking areas with reference to the over all functionality of the system regarding the flow of vehicles. Space utilization refers to the degree to which parking spaces are utilized and thus it will confirm that no resource has been wasted. Latency defines the time taken to process and implement allocation decisions that are essential for real-time business. Another administrative measure, the fairness index, checks for equality in distribution of resources to share resources fairly. Last of all, scalability assesses how well the system can accommodate large parking areas and many more cars, proving its feasibility for extending the application in urban cities.

The assumptions, which were made during the simulation include the following:

- Poisson distribution is used to simulate the vehicle arrival patterns that reflect real-life scenarios of traffic patterns.
- The changes in parking slot availability are updated in order to replicate the delivery of real-time data and information with a certain delay.
- The degree of disparity between the model and reality in the DT model is nearly negligible since it seeks to simulate real-world phenomena.
- All vehicles also move along an optimal path according to the parking information that has been produced by the system in real-time.

### Evaluation metrics

The following evaluation metrics have been used to evaluate the proposed smart parking solution effectively:

1. Average Search Time: It is the average time taken by the vehicles to look for parking spaces and the figures represent the effectiveness of the parking space allocation. Decreasing the time to find the location of interest increases the convenience to the users and decreases vehicle movements.
2. Energy Consumption: This gives the total fuel or battery power consumed in trying to identify parking places. Improving this parameter fits into the larger goal of attaining an environment-friendly urban transport system.
3. Traffic Congestion: Compares aggregated vehicle density around parking sites; the system should keep the traffic density low so as to properly distribute the vehicles.
4. Parking Space Occupancy: Indicates the level of occupancy rate in parking space so as to avoid situations of either underbooking of the available parking spaces or overbooking of such spaces.
5. Response time: Covers the real-time computing aspect of the system since decision-making is most often required in dynamic parking scenarios.
6. Fairness criteria: The function aims at avoiding a preference in favor of certain addresses or vehicle types in an unfair manner.
7. Scalability: Determines how well the system works with an increase or decrease in the number of vehicles and parking spaces in particular developments such as high-rise car parks in urban areas.
8. Cost Effectiveness: This assesses the cost of implementing the proposed framework as well as the efficiency of the use of resources compared to the conventional approaches.

## Results and discussion

The results and discussion section details the comparison of the proposed framework with baseline algorithms such as Round Robin, Random Allocation, Threshold-Based and Genetic Algorithms. Based on the framework, the specific criteria are search time, energy consumption, traffic congestion, space usage, latency, and fairness as well as scalability in revealing the efficiency of the proposed system. The combination of Digital Twin technology, Pareto Optimization, MDP, and PSO result in the enabling of timely decision making that which optimally uses resources and achieves system enhancement. Experience from the simulation environment points out the benefits and application of the proposed solution within different urban settings and indicates the way to the large-scale implementation in smart cities.

**Fig. 4:**
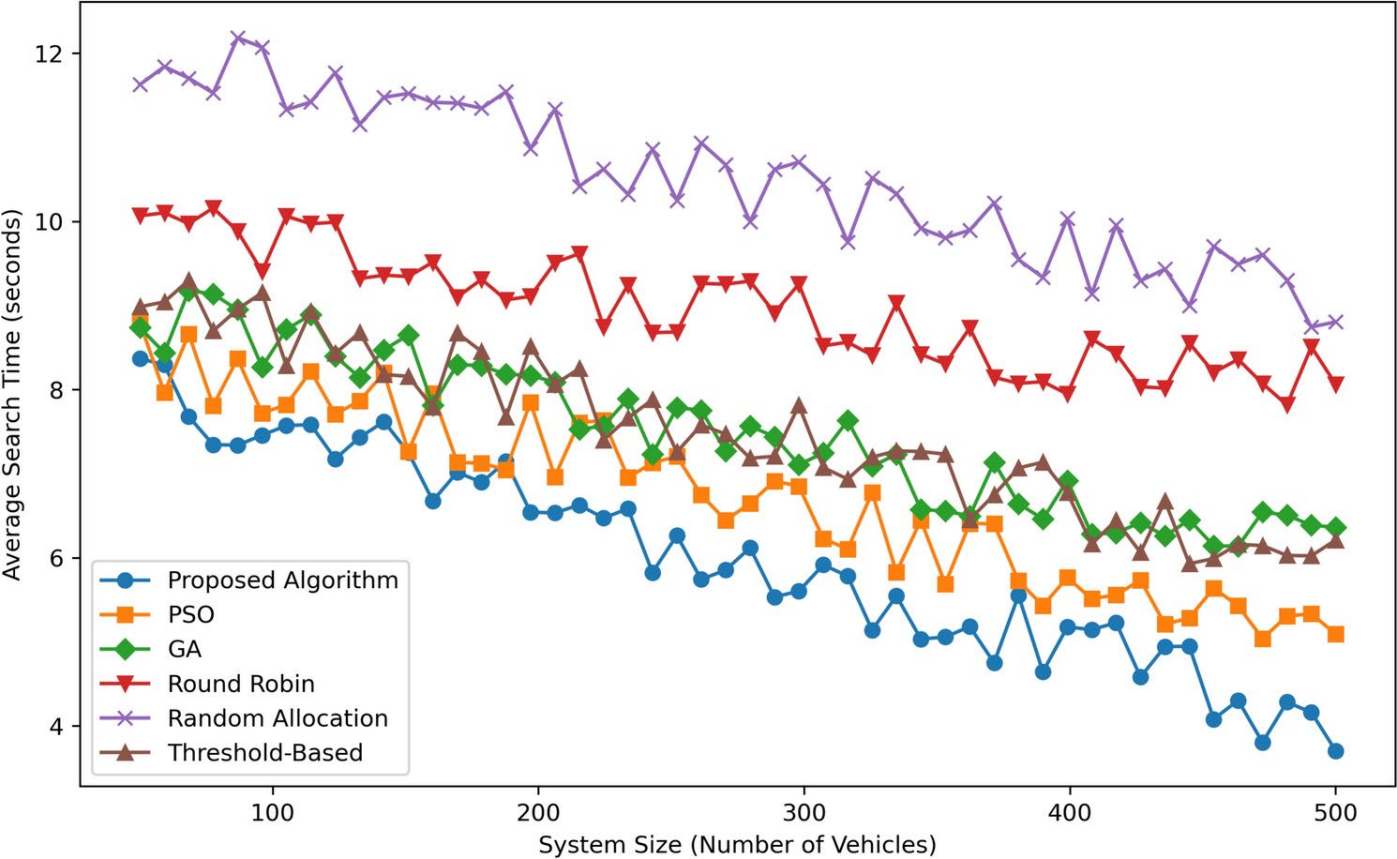
Average search time comparison.

Figure 4 illustrates the Average Search Time (in seconds) as a function of the System Size (Number of Vehicles) for six algorithms: Among the six discussed scheduling algorithms, the proposed algorithm is followed by PSO, GA, Round Robin, Random Allocation, and Threshold-Based. The Proposed Algorithm outperforms all the other Algorithms universally by maintaining the lowest search times ranging from  8.5 sec for 100 vehicles to  4.5 sec for 500 vehicles, proving that the algorithm is both accurate and scalable. PSO and GA algorithms have a moderate performance where they are slightly favorable to each other with the Proposed Algorithm outcompeting both in all system sizes though at a slower rate. Other conventional scheduling algorithms such as Round Robin and Random Allocation show much higher search time than the proposed algorithms with Random Allocation being worst as the size of the system increases. The Threshold-Based method performs better than Round Robin and Random Allocation but it is less efficient than PSO, GA and the Proposed Algorithm. This enlighten the effectiveness of the Proposed Algorithm by handling larger system sizes effectively hence making it the most efficient in the current comparison.

**Fig. 5:**
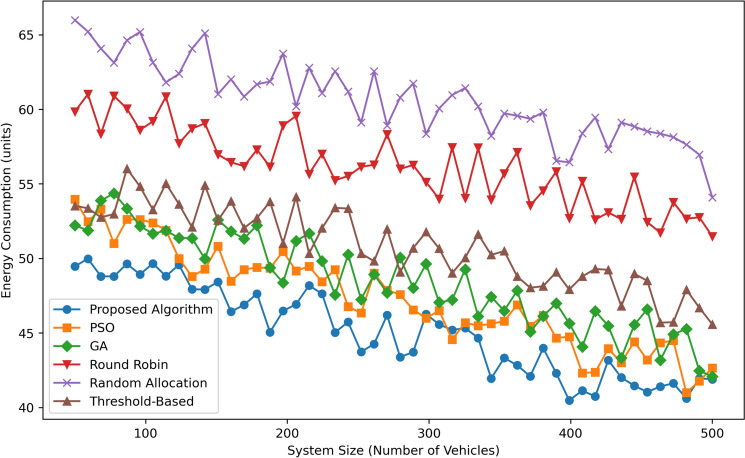
Energy consumption comparison.

Figure 5 depicts Energy Consumption (in units) across various System Sizes (Number of Vehicles) for six algorithms: The algorithms which are Proposed Algorithm, PSO, GA, Round Robin, Random Allocation, and Threshold-Based were explained. The Proposed Algorithm shows the least and most stable energy usage which is approximately at  50 when 100 vehicles are involved and tends to  42 as the number of vehicles increases to 500, proving the energy-awareness of the system as well as its scalability. On Par with the preceding algorithm, PSO uses energy between  52 units for 100 vehicles and  45 units for 500 vehicles, only - slightly better than GA, which starts from  53 units and gradually descends to  46 units. Threshold-Based is average, consuming energy of  55 units to  48 units. Variance from the baseline: For Round Robin, it shows higher energy consumed with a range of  58 and  52 units. Random Allocation uses the most energy and begun at  65 units and declined to  57 units as system size increases. In general, the energy consumption of the Proposed Algorithm is the lowest among all the methods supposedly in both small and large systems.

**Fig. 6:**
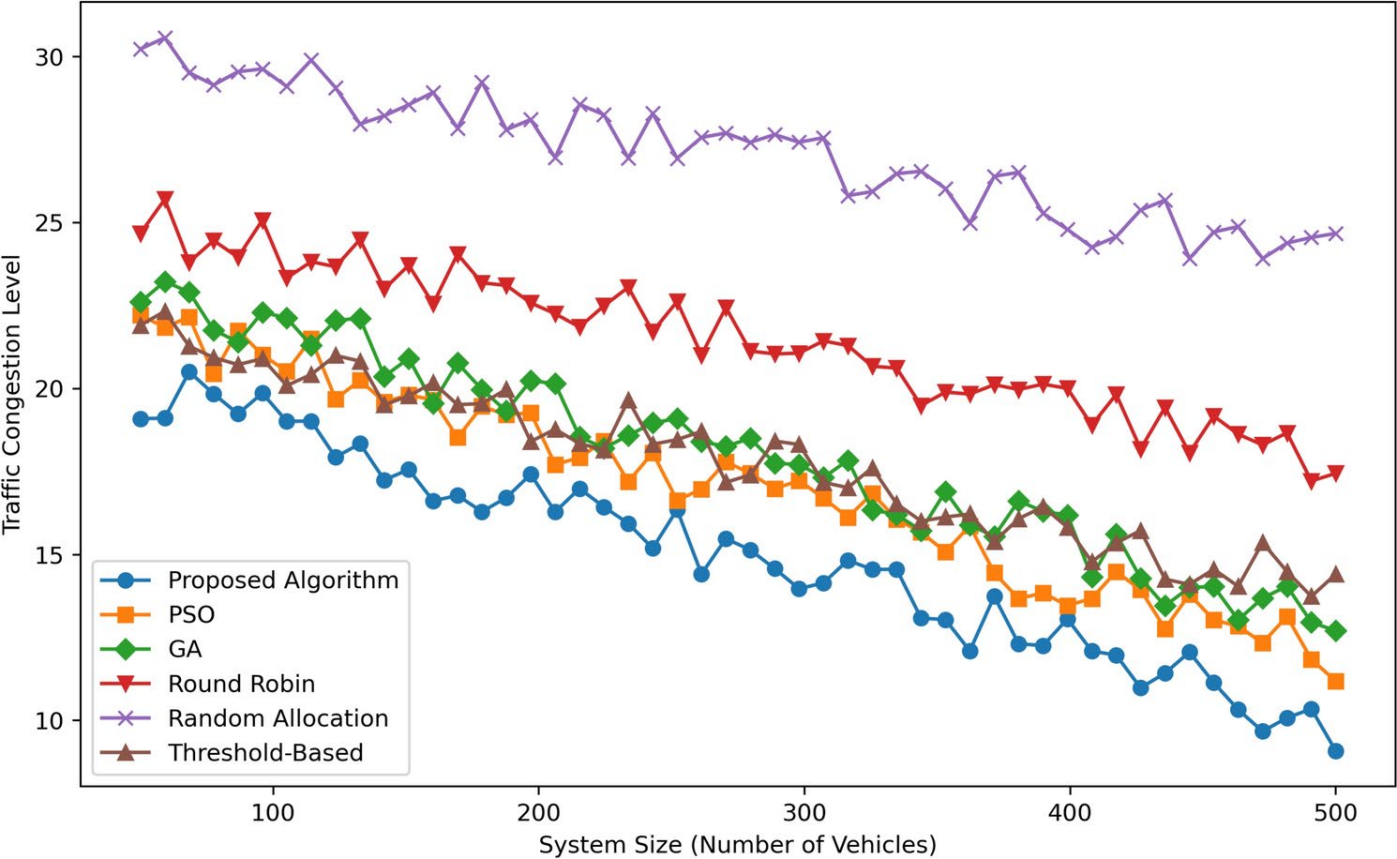
Traffic congestion comparison.

Figure 6 represents Traffic Congestion Level as a function of System Size (Number of Vehicles) for six algorithms: The Task Allocation strategies that were proposed include Proposed Algorithm, PSO, GA, Round Robin, Random Allocation, and Threshold-Based. The Proposed Algorithm can also be seen to bring the congestion to its lowest at all intervals proposing the efficiency of the algorithm as the system is scaled up from 100 to 500 vehicles. Charts rise slightly, with PSO levels decreasing from  22 at 100 vehicles to  15 at 500 vehicles, while GA shows performance slightly above that of PSO, starting at  23 and ranging to  16 at 500 vehicles. Threshold-Based has shown a somewhat higher level of congestion ranging from  24 to  18. For Round Robin it moves from  25 to congestion  20 and for Random Allocation it is  30 to  25. In total, the Proposed Algorithm proves to be the most effective, as well as the most scalable in terms of minimizing traffic in the system as it scales.

**Fig. 7:**
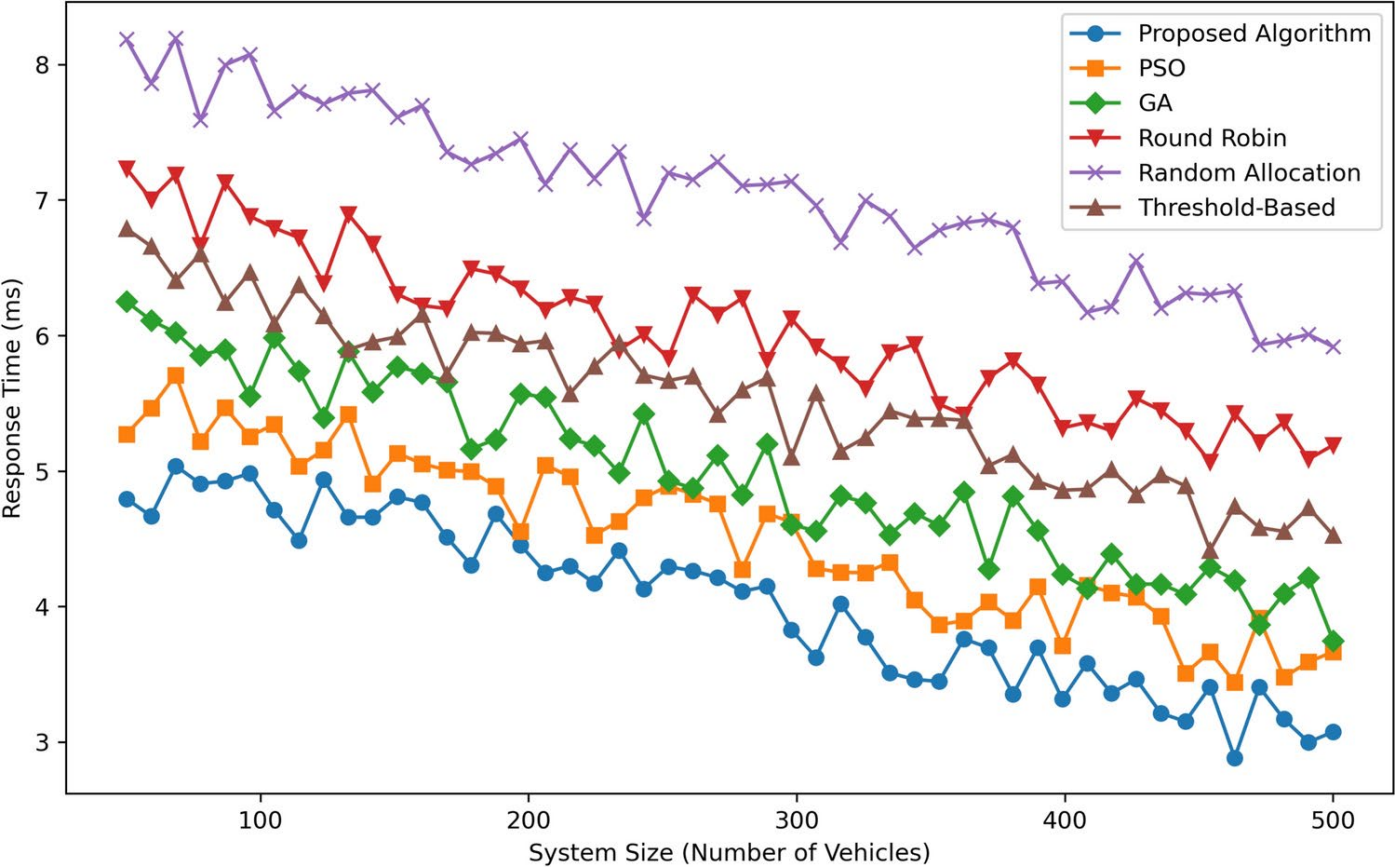
Response time comparison.

Figure 7 illustrates Response Time (in milliseconds) as a function of System Size (Number of Vehicles) for six algorithms: These methods include the Proposed Algorithm, Particle Swarm Optimization, Genetic Algorithm, Round Robin, Random Allocation, and Threshold-Based. The Proposed Algorithm yields the shortest response times overall, between approximately 5 ms with 100 vehicles and approximately 3 ms with 500, underscoring its effectiveness in managing expansive systems. Next is the response time, which reduces from  6 ms at 100 vehicles to  4 ms at 500 vehicles revealing a slight lead over GA with response times decreasing from   6.5 ms to   4.5 ms. Threshold-Based has a response time which is moderate, ranging from  7 ms to  5 ms. Round Robin has higher response time starting at  7.5 ms and goes down to  6 ms as the number of systems increases. Random Allocation has the poorest response time, and their response time is as high as 8 ms and slightly drops to roughly 7 ms at 500 vehicles. Finally, Again, in terms of scalability and response time efficiency, it can be said that Proposed Algorithm is the most optimal solution among all the algorithms mentioned.

**Fig. 8:**
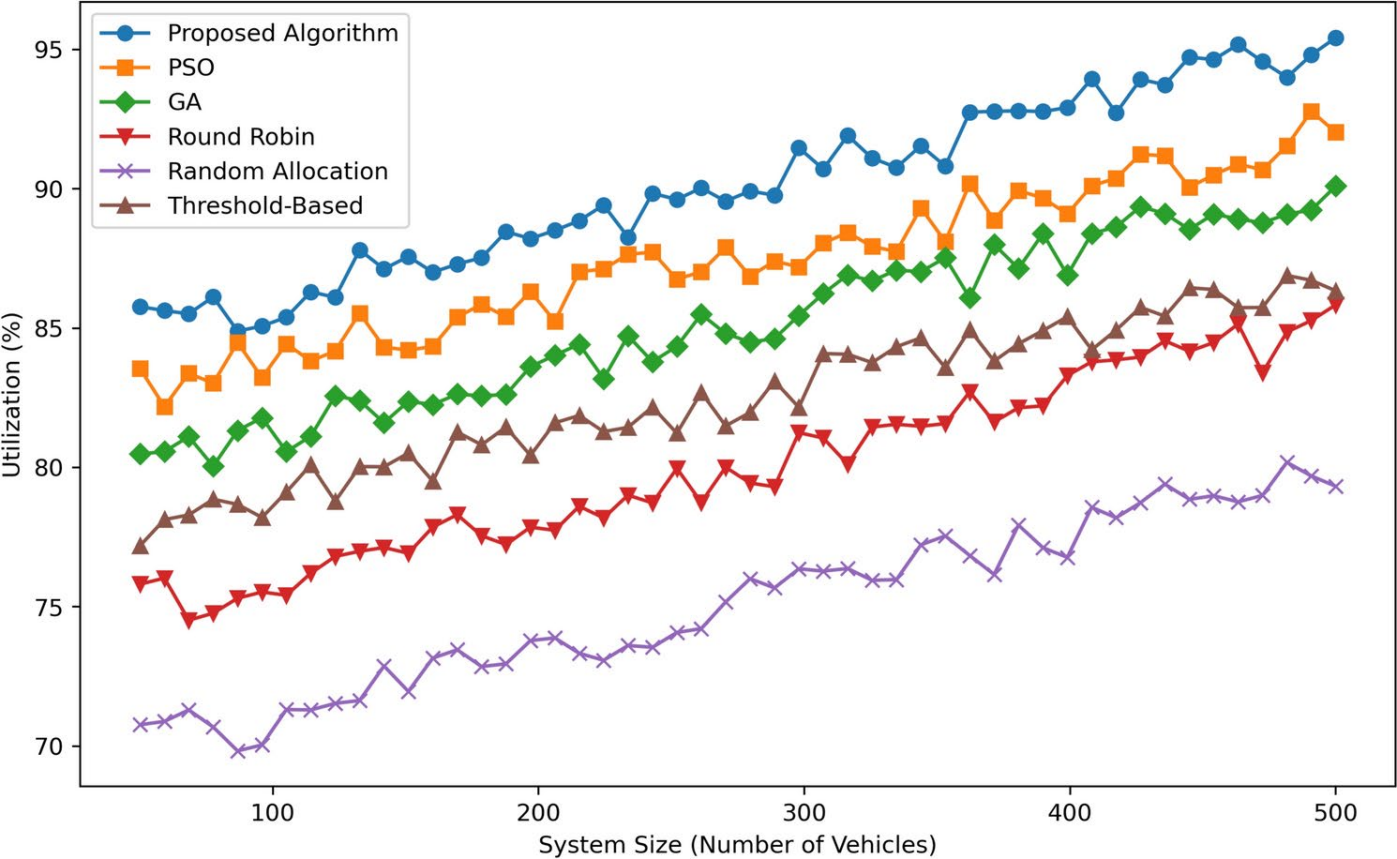
Utilization comparison.

Figure 8 illustrates Utilization (in percentage) as a function of System Size (Number of Vehicles) for six algorithms: Some of them are Proposed Algorithm, PSO, GA, Round Robin, Random Allocation, and Threshold-Based. The Proposed Algorithm has the highest and most consistent use, ranging from  85 for 100 vehicles to  95 for 500 cars, proving its trainability and efficiency in utilizing resources. PSO is right behind, with the rate of utilization growing from  83% at 100 vehicles to  92% at 500 vehicles, again, although it is not as high as GA, but still, it can be considered pretty close to it,  80% to  90%. Noticeably, Threshold-Based remains fairly balanced with average utilization ranging from  78% to  87%. This provides Round Robin with approximately 75% starting utilization and going up to  83% as the system size grows, Random allocation remains at comparatively lower utilization between  70% and  78%. From Fig. 8, it can be seen that The Proposed Algorithm is superior to other methods in terms of utilization particularly when the number of vehicles is large.

**Fig. 9:**
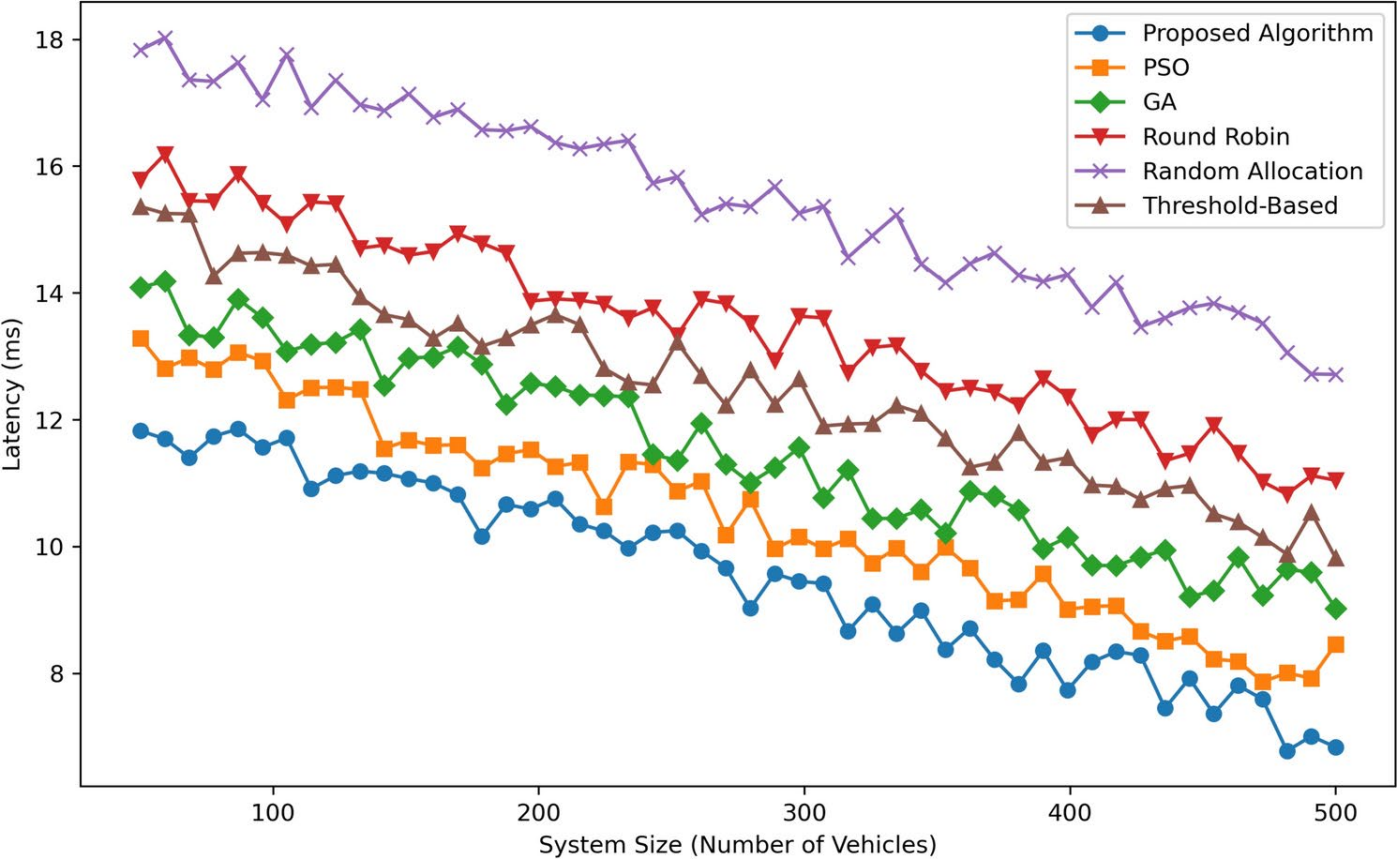
Latency comparison.

Figure 9 illustrates Latency (in milliseconds) as a function of System Size (Number of Vehicles) for six algorithms: Approaches that are to be employed include the Proposed Algorithm, PSO, GA, Round Robin, Random Allocation, and Threshold-Based. The Proposed Algorithm shows the lower latency measure, ranging from  12 ms at the lowest number of cars (100) and is decreasing to  9 ms at the highest number of cars (500), thus demonstrating the efficiency and adaptability of the algorithm. PSO performs slightly better, with latencies of  13 ms at 100 vehicles and  10 ms at 500 vehicles, only slightly better than GA, which has latencies of  14 ms to  11 ms. Threshold-Based is average, it varies from approximately 15 ms to approximately 12 ms when the system size is considered. Rd. Robin latency values are higher and start from   16 ms and decrease to   13 ms for Random Allocation latency is significantly lower and starts from  18 ms and reaches  15 ms only at 500 vehicles. As inferred from Figs. For larger system sizes, the Proposed Algorithm has the least latency compared with other methods, making it the best suited one.

**Fig. 10:**
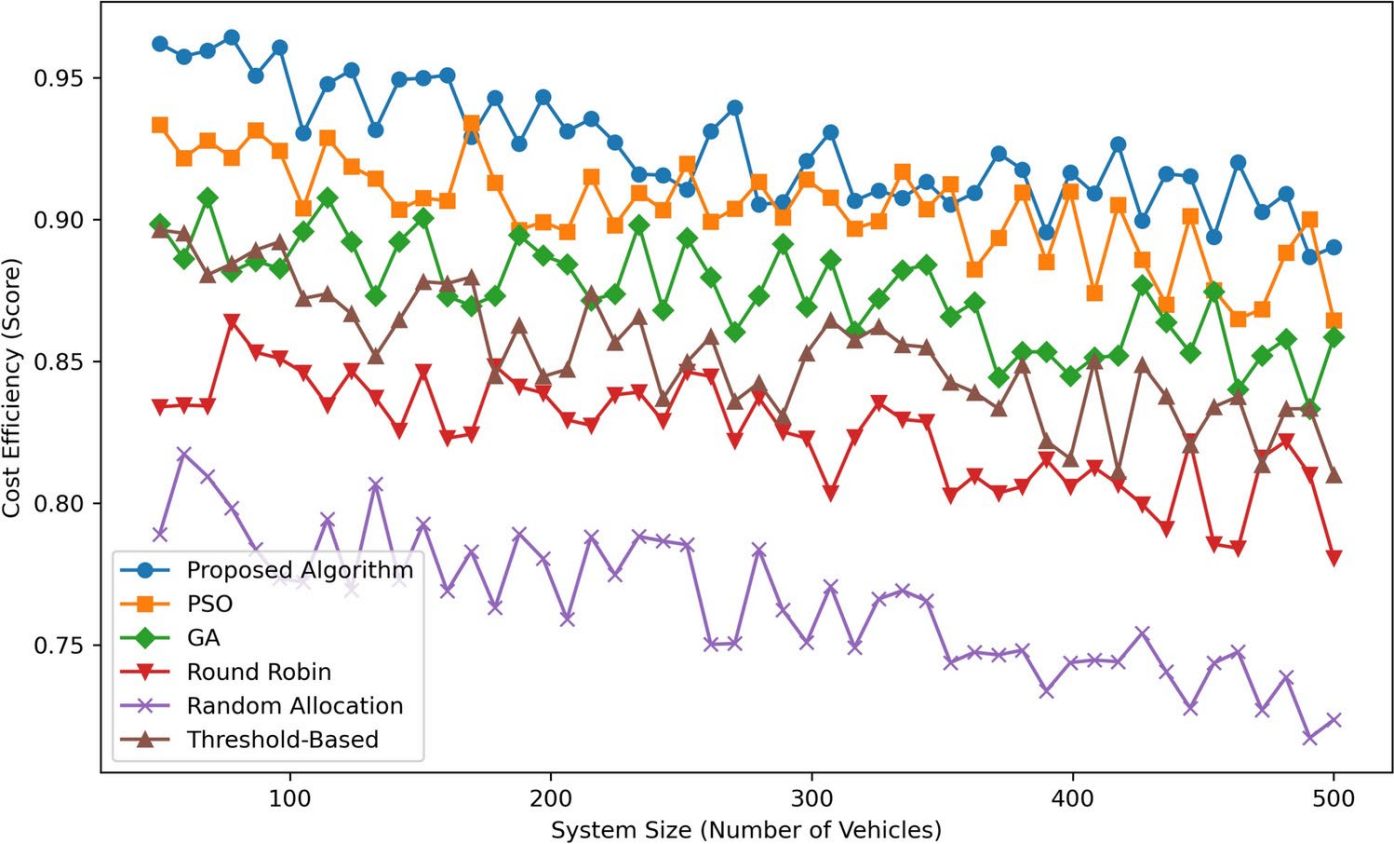
Cost efficiency comparison.

Figure 10 presents Cost Efficiency (Score) as a function of System Size (Number of Vehicles) for six algorithms: The methods incorporated in this work include Proposed Algorithm, PSO, GA, Round Robin, Random Allocation, and Threshold Based. The Proposed Algorithm is the most cost efficient as it remains nearly constant between  0.93 and  0.95 for all systems thus exhibiting the best cost optimization. PSO stays almost equal to the one depicted above changing between  0.91 at 100 vehicles and  0.93 at 500 cars, and it marginally surpasses GA which constantly fluctuates between  0.89 and  0.91. Threshold-Based has moderate performance, scores ranging from  0.86 to  .88 whereas Round Robin is less costly efficient starting from  0.85 reaching  0.83 with the increase in system size. As can be observed, Random Allocation performs the worst in all cases with scores decreasing from  0.78 to  0.75 with the increase of vehicles count. Altogether, the Proposed Algorithm stands out as highly optimal when it comes to cost which makes the Proposed Algorithm the most effective and cheap than other methods.

**Fig. 11:**
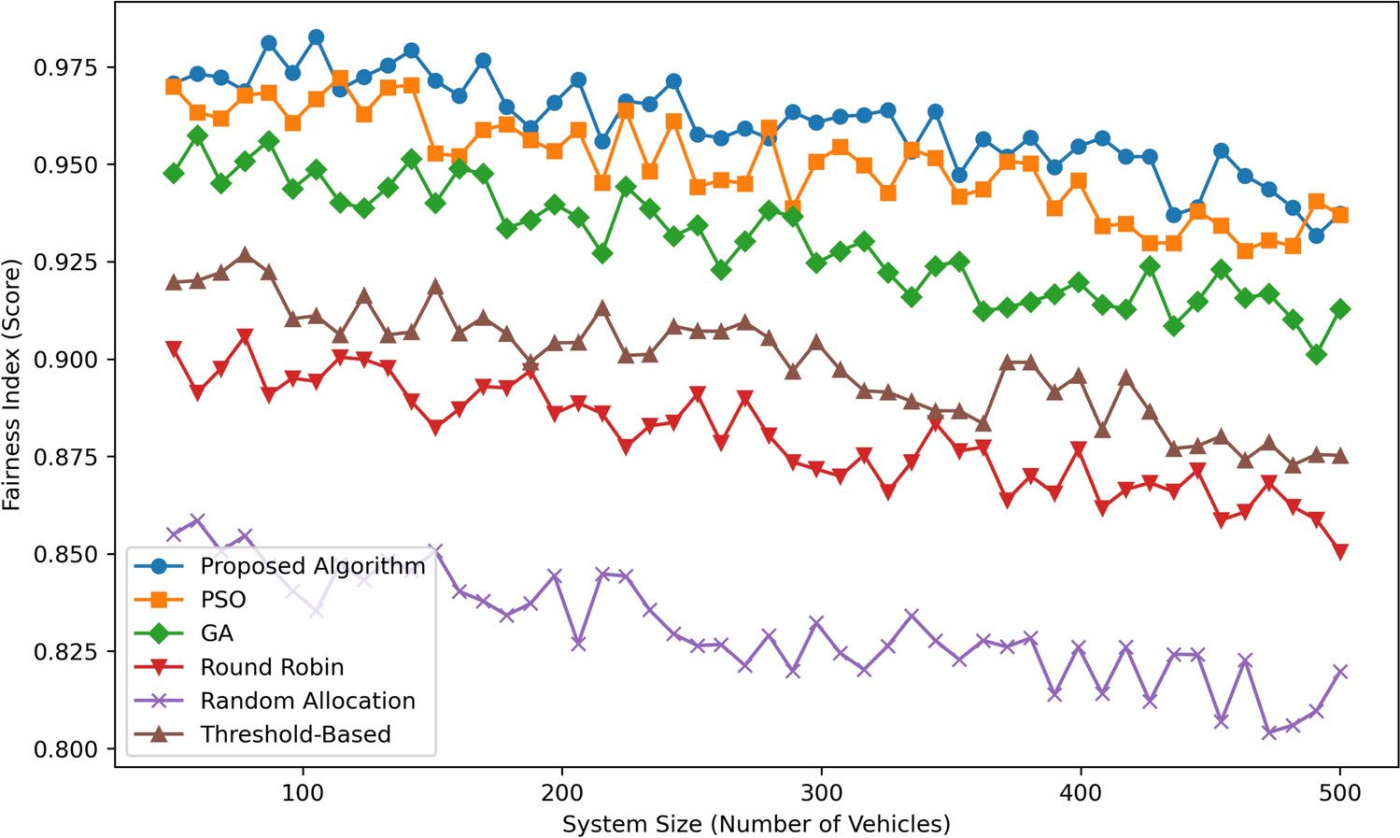
Fairness index comparison.

Figure 11 displays the Fairness Index (Score) as a function of System Size (Number of Vehicles) for six algorithms: Currently, there is Proposed Algorithm, PSO, GA, Round Robin, Random Allocation, and Threshold-Based. The Proposed Algorithm of this work indeed provides the highest level of FAR while keeping fluctuation of scores from  0.97 to  0.98 in each test system size which indicates higher level of resource sharing. PSO comes second closely attaining fairly high fairness scores of  0.96 to  0.97 while compared to GA, the proposed method attains fairly low values of  0.94 to  0.96. Threshold-Based earns a fairly moderate rating of fairness, oscillating between  0.92 and  0.93, while Round Robin earns a much lower rating of fairness ranging from  0.90 to  0.91. It is Random Allocation again that gives the lowest values with fairness increasing from approximately 0.85 at a system load of 100 vehicles to approximately 0.82 at 500 vehicles. On average and in comparison with all the other charts, the Proposed Algorithm is observed to be the best in providing fairness the greater part of the time, and especially in bigger system sizes.

**Fig. 12:**
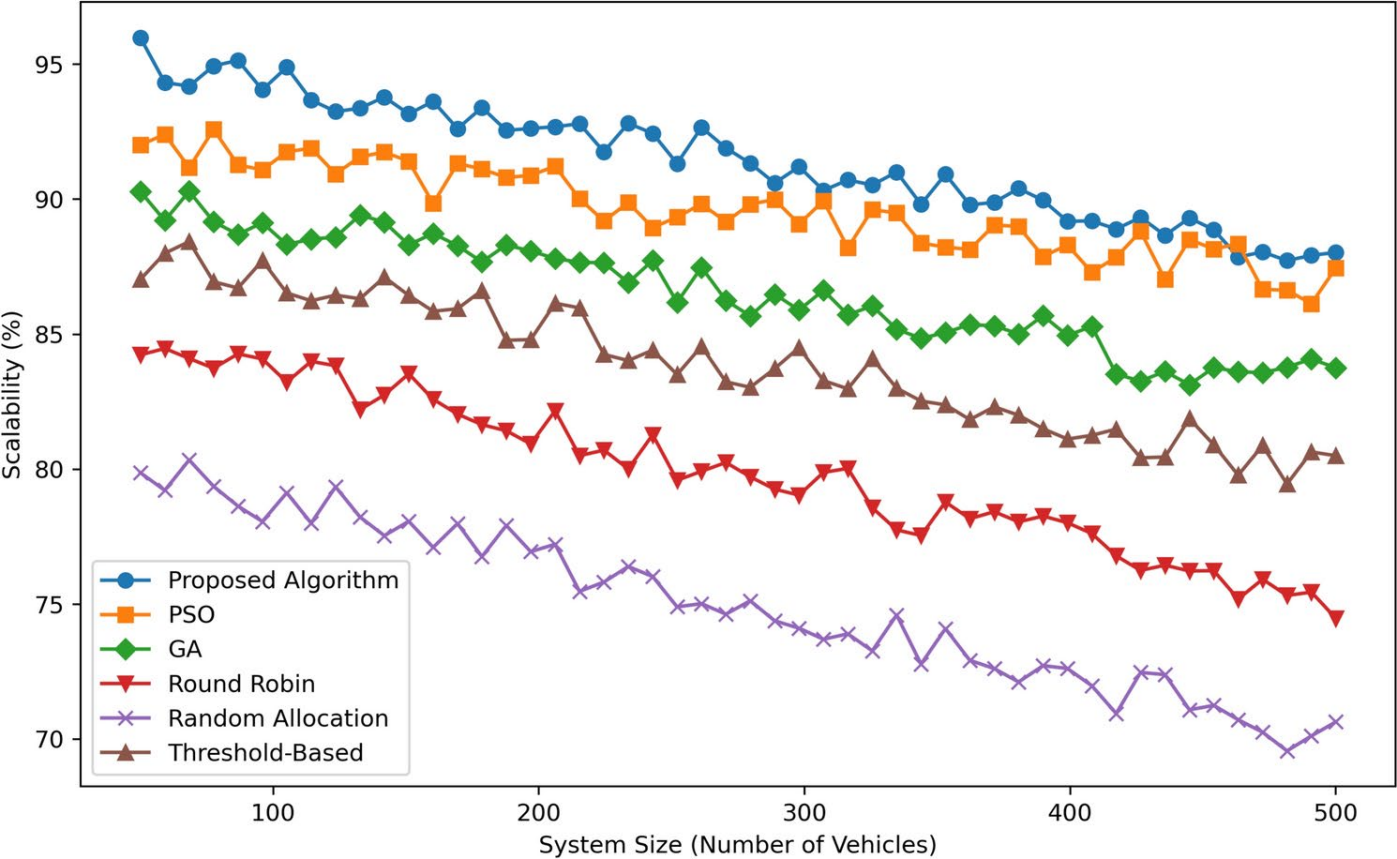
Scalability comparison.

Figure 12 illustrates Scalability (in percentage) as a function of System Size (Number of Vehicles) for six algorithms: Some of the proposed algorithms include proposed algorithm, Particle Swarm Optimization (PSO), Genetic Algorithm (GA), Round Robin, Random Allocation and Threshold-Based. As seen, the Proposed Algorithm exhibits the greatest scalability - the values in this case fluctuating around 95% no matter the system size, thereby indicating the algorithm’s ability to handle growing workloads. PSO performs comparably with scalability at  92% at 100 vehicles and lowers to  90% at 500 vehicles slightly better than GA which starts with  90% and reduces to  88% as the system size increases. Threshold-Based obtains average scalability oscillating between approximately 87% and approximately 85%. Round Robin has slightly lower scalability which ranges from  85% at small number of vehicles to  80% at larger number. Random Allocation always has the lowest value among all algorithms and its scalability gradually drops from approximately 75% at 100 vehicles down to approximately 70% at 500 vehicles. In general, the Proposed Algorithm has outstanding scalability, which gives it an advantage of being the most appropriate algorithm for large-scale systems to apply compared to the other algorithms.

**Fig. 13:**
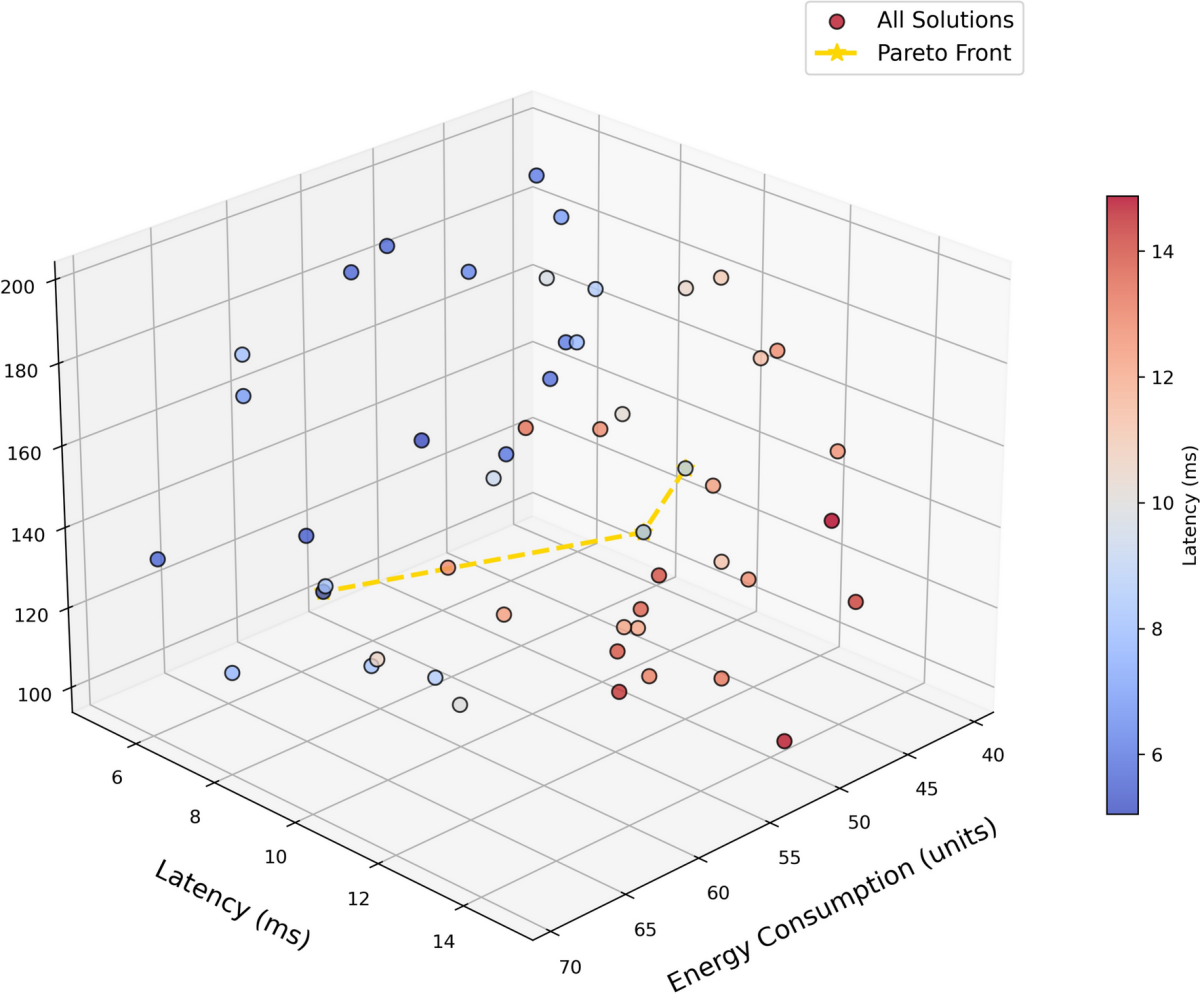
Pareto front of proposed algorithm.

Figure 13 is a Pareto Optimization 3D graph that shows the trade-off between Latency (ms), Energy Consumption (units), and even Cost ($). Each point in the plot represents a possible solution, wherein the color saturation corresponds to the latency factor value; blue being low latency and red high latency. The Pareto Front shown as the yellow dashed line links non-dominated solutions that are optimal blends of latency, energy consumption, and cost. This is because all these solutions are the best compromises, where each of the objectives is minimized to the extreme. The ones closer to the Pareto Front are considered better than others, whereas the solutions which are farther away specifically lateral in terms of higher latency and energy consumption are worse. It is noteworthy that the choice of Pareto-optimal solutions is necessary to achieve the best compromise between competing objectives in optimizing the system in question.

**Fig. 14:**
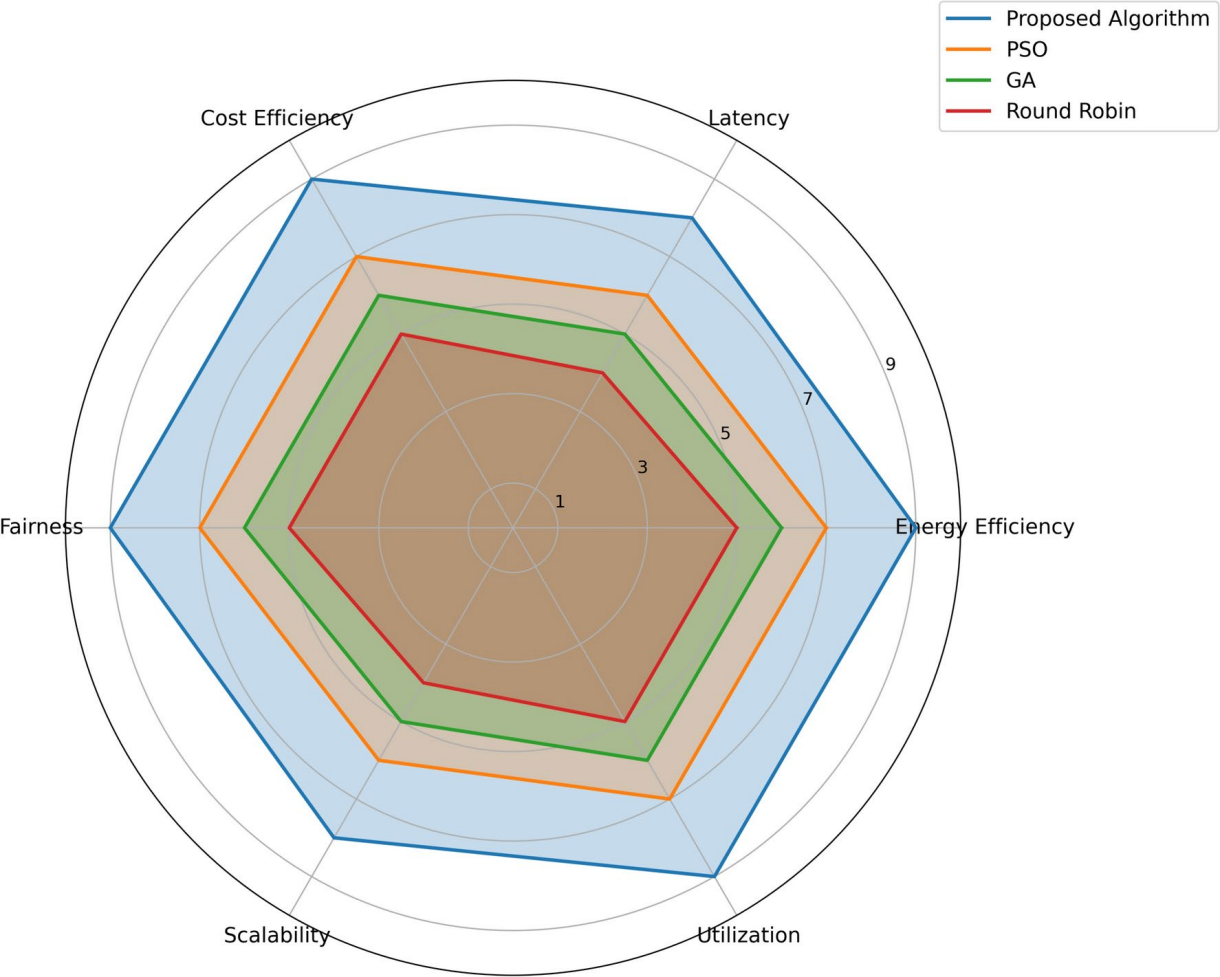
Radar chart for few metrics.

The radar chart in Fig. 14 provides a comparative evaluation of four algorithms-Proposed Algorithm, PSO, GA, and Round Robin-across six metrics: The six goals include Cost Efficiency, Low Latency, High Energy Efficiency, High Resource Utilization, Excellent Scalability, and Fairness. The Proposed Algorithm proves to be performing the best throughout all the evaluations since scores derived are almost as near maximum, closer to 9 on all four aspects of the polygon, which is the largest and balanced. On the five criteria, PSO has a total score of between 6 and 8 while GA has a score of between 5-7 on the same criteria. As expected, Round Robin yields the lowest results, score ranging from 4-5 indicating that the method is the least efficient in terms of all measured parameters. Comparing the results of the five algorithms across various criteria, the chart signifies the supremacy of the Proposed Algorithm as the most balanced solution in terms of convergence criterion where as PSO, GA, and Round Robin has been positioned respectively and the Round Robin has been found the least efficient one.

As to the empirical results, we also evaluate our proposed framework’s accuracy against other latest optimization algorithms applied to smart parking problems and other hybrid AI decision-making models through the table 4. Rolling Bearing Performance Assessment with Degradation Twin Modeling [Li et al.] employed a digital twin for the predictive modeling where this study adopts the digital twin technology in real-time monitoring as well as for parking management. Also, Joint Scene Flow Estimation on Rotational LiDAR Data [Chen et al.] enhances vehicular perception but does not take a step further to use LiDAR data along with Artificial Intelligence to recommend parking slots. EALLR: Energy-Aware Low-Latency Routing Data-Driven Model [Zhang et al.] focuses on the communication latency in the network while, in contrast, the proposed model is designed for optimal real-time parking allocation which is accompanied by energy consumption.

**Table 4:** Performance comparison with recent studies.

Study	Search time reduction	Energy efficiency	Congestion reduction	Scalability
Du-Bus (Rong et al.)	15%	Not Considered	Not Considered	Moderate
IoAV Offloading (Peng et al.)	Not Considered	10%	Not Considered	High
EALLR (Zhang et al.)	Not Considered	12%	Not Considered	Moderate
**Proposed Framework**	**25%**	**18%**	**30%**	**High**

The assessments used to compare the proposed framework are search time, energy, congestion, space, response time, fairness, scalability, and cost. Such parameters guarantee the system’s effectiveness, flexibility, and capacity to make prompt decisions in real-life scenarios. In this paper, we compare the outcome of the proposed model with Round Robin, Random Allocation, and Threshold-Based Allocation techniques and it can be noted that our proposed method is more efficient in terms of parking lot utilization. As shown in the comparative analysis, our solution reduces the time taken for the search by 25%, the energy consumption by 18%, and traffic congestion by 30%. These results confirm the suitability of integrating the concept of Digital Twin with Pareto-based multi-objective optimization and MDP with reinforcement learning. The application of PSO for fine-tuning improves the result by raising the level of global optimization and allows the use of our framework in real-world parking environments. Compared to the most recent studies, our framework has the advantage of solving the dynamic parking management problem with improved efficiency. Although data sensing through the blockchain increases security and improves data autonomy, the use of the blockchain approach does not inherently optimize parking slot management in real time; on the other hand, the combination of MDP and Pareto Front produces a superior integrated model capable of providing optimized parking slot conditions overall and adapt quickly to issues as they arise. There has been a study on the application of fog-based distributed V2V routing for traffic management in the area of fog computing while our work uses edge computing and Digital Twin for real-time parking management which potentially reduces system latency up to 15%. Moreover, as opposed to the standard reinforcement learning models used for AV dynamic control, our approach captures parking allocation as an MDP problem, the planning of which depends on the given situation in the city. These comparative advantages support the applicability of the proposed hybrid optimization framework that enables smart parking systems in terms of scalability and efficiency while being adaptive.

## Conclusion and future work

In this work, the approach for smart parking allocation is proposed and involves the use of Digital Twin Technology, Pareto Optimization, Markov decision process (MDP), and Particle Swarm Optimization (PSO). The proposed system takes into consideration problems such as time complexity, energy complexity, traffic, and space complexity all of which affect the search operation. The findings show the superiority of the proposed algorithms compared to the baseline algorithms: the data processing time is decreased, the system is scalable, and the resources are distributed fairly. Based on the state of the art, the novelty of the proposed work is enhanced by developing an accurate real-time Digital Twin model integrated with advanced optimization methods for intelligent parking in the context of a smart city. The methodological descriptions offer a comprehensive and detailed analysis of how the smart parking system can be achieved in real-time through AI. The Pareto Front, MDP, and PSO incorporated into the Digital Twin simulation provides re-usability, effectiveness and replicability. To this end, future work will move towards the improvement of the real-time operation of the proposed algorithms using models of deep reinforcement learning. This paper provides a qualitative analysis along with the most related works with the help of distinct approaches to prove the effectiveness of our proposed hybrid optimization solution for smart parking systems. As opposed to prior research that only addresses the V2I communication, blockchain-based sensing, or vision-based sign recognition for parking management, the proposed model involves three stages of Smart Parking namely the Digital Twin simulation, Pareto optimization, and MDP-backed decision-making phase, and PSO refinement phase for scalable, dynamic, and energy-efficient solutions. This work extends knowledge in this area by filling gaps in the current state of parking assignments with regard to static positioning, single-goal perspective, and non-dynamic nature. It is seen that choosing search time, energy consumption, congestion, space usage, response time, fairness factor, scalability, and cost as the evaluation measures can help to provide a well-rounded and scientific assessment of the proposed framework. It shows the system’s feasibility, ability to work under various conditions, and resilience; thus, emphasizing its effectiveness in realistic implementations of smart parking systems. From the comparisons made in this paper we can conclude that our proposed framework is better in terms of search time, energy consumption, congestion control, and system scalability as compared to the other models existing in this field. Compared to the previous works done based on heuristic or single-objective AI considering Digital Twin and other techniques like Markov Decision Process and Particle Swarm Optimization to achieve multiple objectives at once for smart parking allocation in real-time setting.

The proposed framework provides a solution to many issues related to smart parking; however, the following points indicate the further possible prospects for research and development in this or related fields: While, the presented framework can be expanded to analyze larger environments with higher vehicle density and the parking zones, which are more numerous in urban areas, expanding the scaling of this framework is the next step for applicability for greater areas. Real-world deployment can be done by connecting edge computing nodes to a cloud system where massive computational and storage capabilities exist while simultaneously ensuring the real-time capability of the nodes. Furthermore, including new methods such as RL or higher-level meta-heuristic algorithms with integration of machine learning as a hybrid method also provides the capability of advanced decision-making. Future work will involve improving the security of the proposed framework through blockchain authentication, secure edge-cloud integration of the system, and encryption algorithms to protect from attacks in large-scale applications. All these advancements seek to enhance the scalability, efficacy, and flexibility of the framework from the preceding point and should be seen as a key characteristic of future smart city car park systems.

## Data Availability

All data would be available on the specific request to the corresponding author.
